# Long Chain Polyunsaturated Fatty Acids Docosahexaenoic Acid and Arachidonic Acid Supplementation in the Suckling and the Post-weaning Diet Influences the Immune System Development of T Helper Type-2 Bias Brown Norway Rat Offspring

**DOI:** 10.3389/fnut.2021.769293

**Published:** 2021-11-01

**Authors:** Dhruvesh Patel, Marnie Newell, Susan Goruk, Caroline Richard, Catherine J. Field

**Affiliations:** Department of Agricultural, Food and Nutritional Science, University of Alberta, Edmonton, AB, Canada

**Keywords:** nutritional immunology, immune development, T helper type-2 rodent model, infant nutrition, neonatal development, suckling diet, post-weaning diet, immunomodulatory effect

## Abstract

**Background:** Dietary long chain polyunsaturated fatty acids (LCPUFA) such as arachidonic acid (ARA) and docosahexaenoic acid (DHA) play an important role in the development of the infant immune system. The role of LCPUFA in the T helper type 2 (Th2) biased immune system is unknown. We aimed to understand the effect of feeding LCPUFA during suckling and post-weaning on immune system development in Th2 bias Brown Norway rat offspring.

**Methods:** Brown Norway dams were randomly assigned to nutritionally adequate maternal diet throughout the suckling period (0–3 weeks), namely, control diet (0% ARA, 0% DHA; *n*= 8) or ARA + DHA (0.45% ARA, 0.8% DHA; *n* = 10). At 3 weeks, offspring from each maternal diet group were randomized to either a control (0% ARA, 0% DHA; *n* = 19) or ARA+DHA post-weaning (0.5% ARA, 0.5% DHA; *n* = 18) diet. At 8 weeks, offspring were killed, and tissues were collected for immune cell function and fatty acid composition analyses.

**Results:** ARA + DHA maternal diet resulted in higher (*p* < 0.05) DHA composition in breast milk (4×) without changing ARA levels. This resulted in more mature adaptive immune cells in spleen [T regulatory (Treg) cells and B cells], mesenteric lymph nodes (MLN, lower CD45RA+), and Peyer's patches (PP; higher IgG+, B cells) in the ARA+DHA group offspring at 8 weeks. ARA+DHA post-weaning diet (3–8 weeks) resulted in 2 × higher DHA in splenocyte phospholipids compared to control. This also resulted in higher Th1 cytokines, ~50% higher TNF-α and IFNγ, by PMAi stimulated splenocytes *ex vivo*, with no differences in Th2 cytokines (IL-4, IL-13, and IL-10) compared to controls.

**Conclusion:** Feeding dams a diet higher in DHA during the suckling period resulted in adaptive immune cell maturation in offspring at 8 weeks. Providing ARA and DHA during the post-weaning period in a Th2 biased Brown Norway offspring model may support Th1 biased immune response development, which could be associated with a lower risk of developing atopic diseases.

## Introduction

Docosahexaenoic acid (DHA), an omega-3 (*n*−3/ω-3) long chain polyunsaturated fatty acid (LCPUFA), and arachidonic acid (ARA), an *n*−6 LCPUFA are essential fatty acids needed for infant neural and vision development (1, 2). However, their role in the development of the immune system, particularly in those at risk for atopic conditions due to T helper type-2 (Th2) dominant immunity, has been sparsely studied. Studies have shown beneficial effects of DHA in populations predisposed to developing conditions associated with Th2 dominance (3, 4). However, these studies were often conducted using a diet containing no ARA or a very high level of DHA. Such dietary conditions have anti-inflammatory and immunosuppressive effects (5, 6) and may result in a lower concentration of ARA in the immune cells (7, 8).

The essentiality of ARA in the formula for development in a term infant has been debated despite its presence in human milk (9, 10). The importance of ARA supplementation, alongside DHA, on infant immune system development has been shown in healthy term infants by our group (11). Previous studies from our lab with DHA and ARA supplementation, conducted in infants and Sprague-Dawley rats, have shown beneficial effects of ARA+DHA on the immune response and oral tolerance development (12–15). Yet to date, no studies have assessed the combination of dietary ARA + DHA in a Th2 biased preclinical model.

Passive immunity in neonates arises during pregnancy and lactation, contributing to the initial immune response against pathogens (16). This changes as the immune organs develop and the adaptive immune system matures in an infant. In the first few months after birth, neonates rely heavily on the innate immune system, consisting of granulocytes, antigen presenting cells (APC), macrophages, and natural killer (NK) cells to protect against pathogens until the adaptive immune response, through T cells and B cells, reaches a more adult level of maturation (17). T cells and B cells first enter the bloodstream from the primary lymphoid organs (thymus and bone marrow, respectively), in a naïve state and are unable to mount an effective response toward pathogens. Therefore, they undergo additional education in the secondary lymphoid organs (such as spleen and lymph nodes) to become effector cells that are able to mount an adult-level of immune response to pathogens (18). Similarly, gut associated lymphoid tissue (GALT), including Peyer's patches (PP) and mesenteric lymph nodes (MLN), form germinal centers and result in rapid changes in adaptive immune cell populations post-weaning when the intestine is exposed to solid-food derived antigens (19, 20). Therefore, the development of the spleen, lymph nodes, and GALT becomes critical for the maturation of adaptive immunity (21). Furthermore, the nutrition provided during these periods is a source of nutrients and antigens necessary for adaptive immune cell maturation (22).

The immune response during early infancy is skewed toward a prenatally derived Th2 cytokine pattern, such as IL-4, IL-5, and IL-13, making them more susceptible to infection and allergic reactions (17, 23, 24). As infants are introduced to solid food between 6 and 12 months of age, they rely less on maternally derived passive immunity and develop antigen-specific adaptive immunity which is associated with an increase in Th1 cytokines (IL-2, IFNγ, TNF-α) and cell mediated immune responses (25). If the switch from Th2 (characterized by a high level of Th2 cytokines and low level of Th1 cytokines) to Th1 does not occur, it can lead to conditions such as food allergies and atopic disorders (26). The Th2 dominant environment of Brown Norway rats makes them highly prone to developing food allergies with high levels of Immunoglobulin-E (IgE) and IgG1 antibodies (Th2 related antibodies) (27). More specifically, they have higher circulating CD45RC^low^CD4+ T cells and lower circulating CD8+ T cells resulting in fewer Th1 cytokines and more Th2 cytokines (IL-4 and IL-13) compared to other rodent models upon *ex vivo* stimulation with T cell mitogens (27, 28). This dominant Th2 environment provides a unique condition, similar to infants with a genetic predisposition to allergies, to study the immunomodulatory properties of LCPUFA on immune system development (29). However, LCPUFA supplementation during early infancy on immune system development has not been assessed. Therefore, the primary objective of the current study is to determine the effect of feeding ARA+DHA during the suckling period (through the maternal diet), and in the post-weaning period on (1) LCPUFA status and (2) immune system development and function in Brown Norway offspring at 8 weeks. We hypothesized that the addition of ARA and DHA to their diet would be beneficial for the Th2 bias Brown Norway offspring by promoting a Th1 response and early development of adaptive immune cells.

## Materials and Methods

### Study Design and Diet

Experimental diets were isocaloric, isonitrogenous, and nutritionally adequate. The macronutrient and micronutrient composition of the semi-purified basal diet (fat omitted) used has been previously described in detail (30). The fat mixture added to the experimental diets (20/100 g diet) was obtained by blending lard, olive oil, Mazola canola oil, Mazola corn oil, ARAsco, and DHAsco (DSM, Nutritional Products, Columbia, MD, USA). The experimental diets were closely matched to have similar PUFA to saturated fatty acid (SFA) ratios and n-6 to n-3 ratios (Table 1). The diets were prepared biweekly and stored at 4°C until used to prevent exposure to air.

**Table 1:** Total fatty acid composition of the experimental maternal diets fed during the suckling period and post-weaning diets fed to Brown Norway offspring^a^.

**Fatty acid (g/100 g**	**Control**	**ARA + DHA**	***P*-maternal**	**Control post-weaning**	**ARA + DHA**	***P*-Post-Weaning**
**total fatty acids)**	**maternal diet**	**maternal diet**	**diet^*c*^**	**post-weaning diet**	**post-weaning diet**	**diet^*d*^**
14:0	0.9 ± 0.1	1.2 ± 0.1	0.11	0.9 ± 0.1	1.0 ± 0.1	0.50
16:0	19.5 ± 0.2	20.7 ± 0.2	0.03	19.5 ± 0.2	19.9 ± 0.6	0.52
18:0	10.6 ± 0.1	11.3 ± 0.2	0.07	10.6 ± 0.1	11.0 ± 0.4	0.36
16:1*n*−7	1.6 ± 0.1	1.7 ± 0.1	0.38	1.6 ± 0.1	1.6 ± 0.1	0.95
18:1*n*−9	43.9 ± 1.0	39.4 ± 1.5	0.08	44.0 ± 1.0	42.7 ± 2.6	0.62
18:2*n*−6 LA	19.9 ± 0.3	21.3 ± 0.5	0.07	19.9 ± 0.3	19.7 ± 0.7	0.78
20:4*n*−6 ARA	0.0 ± 0.0	0.5 ± 0.1	<0.001	0.0 ± 0.0	0.5 ± 0.0	<0.001
18:3*n*−3 ALA	2.5 ± 0.0	1.9 ± 0.0	0.001	2.5 ± 0.0	2.1 ± 0.1	0.01
22:6*n*−3 DHA	0.0 ± 0.0	0.8 ± 0.0	<0.001	0.0 ± 0.0	0.6 ± 0.1	<0.001
Total^b^ SFA	31.1 ± 0.1	33.2 ± 0.1	0.002	31.1 ± 0.1	31.9 ± 1.2	0.40
Total PUFA	22.4 ± 0.3	24.5 ± 0.6	0.04	22.3 ± 0.3	22.8 ± 0.9	0.63
Total MUFA	45.6 ± 0.9	41.1 ± 1.4	0.07	45.5 ± 0.9	44.3 ± 2.5	0.61
Total *n*−6	19.9 ± 0.3	21.8 ± 0.5	0.04	19.9 ± 0.3	20.7 ± 0.7	0.70
Total *n*−3	2.5 ± 0.0	2.7 ± 0.0	0.03	2.5 ± 0.0	2.6 ± 0.2	0.36
Ratio *n*−6/*n*−3	8.1 ± 0.0	8.1 ± 0.1	0.65	8.1 ± 0.1	7.8 ± 0.2	0.18

a *Fatty acid analysis of diet was done using gas liquid chromatography. Data are presented as the mean ± SEM of 2–3 batches of oil mix used for diet. ALA, α-linolenic acid; LA, linoleic acid; ARA, arachidonic acid; DHA, docosahexaenoic acid; PUFA, polyunsaturated fatty acid; MUFA, monounsaturated fatty acid; SFA, saturated fatty acid*.

b *Some fatty acids that were traced were not presented as they were very small or not relevant, this may lead to a mismatch in total proportions of fatty acids such as total SFA, PUFA, MUFA, n−6, and n−3*.

c *Indicates p-value for a T-test for the difference between maternal diets*.

d *Indicates p-value for a T-test for the difference between post-weaning diets*.

**Figure 1:**
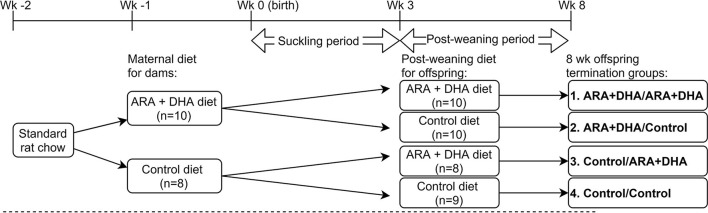
Animal study design. Pregnant Brown Norway dams were assigned to either arachidonic acid + docosahexaenoic acid (ARA + DHA) supplemented maternal diet (*n* = 10) or control diet (*n* = 8) 5–7 d before parturition and continued the same diet throughout the suckling period (3 weeks). Male and female offspring were culled between the same diet group of dams to get an approximate sex ratio match between the 4 diet groups. Offspring breastfed to their dam during the suckling period. At the end of the suckling period, offspring from each dam consumed either ARA + DHA post-weaning diet or control post-weaning diet until 8 weeks (post-weaning period) from birth. Dams were killed at the end of the suckling period and offspring at the end of the post-weaning period. The animal experiments were conducted in serial blocks to achieve *n* = 8 offspring per diet group.

Animal care and experiments were conducted as per guidelines stipulated by the Canadian Council on Animal Care and approved by the University of Alberta Animal Ethics Committee (AUP00000125). Timed-pregnant Brown Norway rats (Charles River Laboratories, Laval, Quebec, Canada; *n* = 18) were obtained on day 7 of gestation and housed in a temperature and humidity-controlled environment with a 12/12 h reverse light cycle. Dams were fed standard rat chow purified diet (Lab diet 5001; PMI Nutrition International, St Louis, MO, USA) for 1 week acclimatization period (Figure 1). One week prior to parturition, dams were randomized to consume one of the experimental diets: ARA+DHA (0.45% ARA, 0.8% DHA w/w of total fat; *n* = 10) or control (0% ARA, 0% DHA; *n* = 8) diet (Table 1). The DHA concentration for the maternal diet was selected to achieve the upper end (0.8% of total fat as DHA) of breastmilk reported in human populations (31). Subsequently, the ARA concentration was selected to attain an ARA/DHA ratio of 1:2, as previous experiments from our lab found this ratio effective in increasing DHA levels without affecting the ARA levels in immune cell phospholipids. The litters were culled, meaning the pups were cross-fostered to ensure an equal number of offspring per dam (3–5 pups/dam) to avoid weight differences in the offspring. Dams were fed experimental diets *ad libitum* throughout the suckling period (3 weeks). At the age of 3 weeks, offspring from each dam were randomized to either ARA+DHA post-weaning diet (0.5% DHA and 0.5% ARA w/w of total fat, *n* = 18) or control post-weaning diet (0% ARA and 0%DHA, *n* = 19) until 8 weeks of age. The DHA concentration of 0.5% of total diet fat was calculated based on physiologically achievable levels of DHA with 2–3 servings of fish per week, as outlined in the 2015–2020 dietary guideline for Americans (Eighth edition) (32) and the ARA concentration of 0.5% was selected to obtain a 1:1 ratio for ARA/DHA. Male and female offspring were equally distributed between post-weaning diet groups to enable the studying of sex effects. The offspring had unrestricted access to food and water. Food cups were changed every 2–3 days to limit air exposure. Bodyweight and food intake were regularly recorded. Dams were killed at the end of the 3-week suckling period and all the offspring were euthanized at 8 weeks (end of post-weaning period) and tissues were collected.

### Tissue Collection: Spleen, MLN, PP, Blood and Plasma, and Mammary Tissue

Following euthanization, blood was collected by cardiac puncture, centrifuged (1,734 × g; 10 min; 22°C), plasma aliquoted, and stored at −80°C until further analysis. The spleen, MLN, and PP from the offspring and the mammary gland from the dams were aseptically removed. Immune cells from the spleen, MLN, and PP were isolated as previously described (33). Briefly, tissues were pushed through a nylon mesh screen to obtain a single cell suspension. Erythrocytes were lysed with ammonium chloride lysis buffer (155 mM NH_4_Cl, 0.1 mM EDTA, and 10 mM KHCO3; Fisher Scientific, Alberta, Canada), and cells were washed and resuspended in complete cell culture media (RPMI 1640 media supplemented with 5% v/v fetal calf serum, 2.5 mM 2-mercaptoethanol, and 1% antibiotic / antimycotic; Thermo Fisher Scientific, Mississauga, Ontario, Canada). Cell concentration was determined using cell viability trypan blue dye (Sigma-Aldrich) and hemocytometer and diluted to 1.25 × 10^6^ cells/ml. Note, we were not able to collect cells at the required concentration from MLN and PP for some 8-week offspring. Therefore, the final group size assessed for fatty acid analysis and immune cell phenotyping differs from the original group size and is detailed in the results.

### *Ex vivo* Immune Cell Stimulation and Cytokine Measurement

*Ex vivo* cytokine production by stimulated immune cells was measured as previously described (34). Briefly, spleen or MLN immune cells (1.25 × 10^6^ cells/ml) were incubated in the presence or absence of phorbol-myristate-acetate and ionomycin (PMAi, 2 μl/ml, Cell Stimulation Cocktail, Thermo Fisher Scientific, Mississauga, Ontario, Canada), and lipopolysaccharide (LPS, 2 μl/ml, Thermo Fisher Scientific, Mississauga, Ontario, Canada) for 72 h at 37°C and 5% CO_2_. Cells were then centrifuged, and supernatant collected and stored at −80°C. Commercial ELISA kits were used to measure cytokine concentration (range of detection in brackets) of IL-1β (15.6–4,000 pg/ml), IL-2 (250–4,000 pg/ml), IL-6 (31.3–4,000 pg/ml), IL-10 (15.6–4,000 pg/ml) and TNF-α (31.3–4,000 pg/ml) (R&D Systems, Minneapolis, MN, USA), IFN-γ (8–1,024 pg/ml, U-CyTech Bioscience, Cedarlane, Burlington, Ontario, Canada), TGF-β1 (31.3–500 pg/ml, BioLegend, San Diego, CA, USA), and IL-4 (0.1–4,500 pg/ml) and IL-13 (0.1–3,200 pg/ml) (Mesoscale Discovery, U-Plex, Rockville, MD, USA) as per the instruction of the manufacturer. Absorbance was read on a spectrophotometer, and concentrations were calculated using a standard curve (SpectraMax 190 Microplate Reader, Molecular Devices, San Jose, CA, USA). All measurements were conducted in duplicate with a CV <15%.

### Fatty Acid Analysis of Phospholipid and Total Lipids

Total lipids from the diet, mammary gland, splenocytes, MLN, and plasma were extracted by modified Folch as previously described (12, 35). Total phospholipids from splenocytes, MLN, and plasma were further isolated by thin layer chromatography on silica G plates and fatty acids separated by automated gas liquid chromatography (Agilent Technologies, Ontario, Canada) on a 100 m CP-Sil 88 fused capillary column as previously described (36). They were quantified as relative percent of total phospholipid fatty acid content.

### Immune Cell Phenotype

The phenotype of isolated immune cells from the spleen, MLN, and PP was assessed by direct immunofluorescence assay as previously described (37). Four-color flow cytometry was used to identify cell surface markers in combinations. The fluorochrome of monoclonal antibodies (mAb) was selected in a way to minimize or avoid spectra overlap, and compensation controls and unstained controls for each fluorochrome were used to guide the gating strategy. For splenocytes, the following combination of mAb were used: CD3/CD25/CD4/CD8, CD25/CD3/FoxP3/CD4, TCRαβ/CD27/CD8/CD4, CD4/CD152/CD8/CD28, CD25/OX62/OX6/CD11, CD284/CD68/CD11/CD45RA, OX12/CD86/OX6/CD45RA, CD3/CD161, IgE/CD45RA/IgG, and IgA/CD45RA. For immune cells of MLN, the following combinations of monoclonal antibodies were used: CD3/CD25CD4/CD8, CD25/CD3/FoxP3/CD4, TCRαβ/CD27/CD8/CD4, CD4/CD152/CD8/CD28, OX12/CD27/OX6/CD45RA, IgA/CD45RA/IgG, and IgE/CD45RA. For immune cells of PP, the following combinations of monoclonal antibodies were used: CD3/CD25CD4/CD8, CD284/CD68/CD11/CD45RA, OX62/CD86/OX6/CD45RA, IgA/CD45RA/IgG, and IgE/CD45RA. Note that CD152 identifies CTLA-4 on T cells, OX62 marker identifies the integrin molecule on dendritic cells, OX6 identifies MHC-II on antigen presenting cells, CD68 identifies tissue resident macrophages, and OX12 identifies Ig-κ chain on splenic B cells. All antibodies were purchased from Biolegend (San Diego, CA, USA) or BD Biosciences (Mississauga, Ontario, Canada). Briefly, immune cells (2,00,000) were incubated for 30 min at 4°C with cell surface monoclonal antibodies. Subsequently, for intracellular staining of FoxP3, cells were fixed, permeabilized, and stained with fluorophore-conjugated FoxP3 antibody for 20 min. Then, the cells were washed, fixed in paraformaldehyde (10 g/L; Thermo Fisher Scientific, Mississauga, Ontario, Canada), and acquired within 72 h by flow cytometry (FACSCalibur; Becton-Dickinson, San Diego, CA, USA) according to the relative fluorescence intensity and analyzed using FlowJo software. The gating strategies for major cell types are described in Supplementary Figure 1.

### Statistical Analysis

Data are presented as mean ± SEM unless stated otherwise. The current study was powered to assess the primary objective of immune function and the secondary objective of changes in the fatty acid composition of various tissues in the Th2 biased Brown Norway rats. The sample size of *n* = 8 per group was calculated based on experiments conducted in our lab using healthy Sprague-Dawley rats, in which a difference of 20% (β-value) could be identified at a significance level of 5% (α-value). Data were checked for normality and homogeneity. When the assumptions criteria were not met, the data were transformed, and assumptions were rechecked before conducted statistical analysis. Data were analyzed using the Proc Mixed procedure 3-factor ANOVA with suckling (maternal) diet, post-weaning diet, and sex as the main effects (SAS 9.4 software, Cary, NC, USA). However, only a two-factor ANOVA was pursued when sex or an interaction effect involving sex was not statistically significant. This allowed us to use a 2 × 2 study design to study the effect of suckling diet, post-weaning diet, and suckling diet × post-weaning diet interaction. Note, when a significant interaction between suckling diet × post-weaning diet was observed, a *post-hoc* analysis (PROC LSMEANS) was conducted to compare means of the four groups (ARA+DHA/ARA + DHA, ARA + DHA/Control, Control/ARA + DHA, and Control/Control). Furthermore, when there was a trend toward significance, the four groups based on diet were reported for better visualization. When the post-weaning diet variable was absent (fatty acid analysis of diet and mammary gland for dams), an unpaired Student's *t*-test was conducted for 2 group comparisons. Differences were assumed to be significant at *p* ≤ 0.05 (two-tailed).

## Results

### Growth Parameters

There were no significant effects of the suckling diet or post-weaning diet on the body weight (mean 156 ± 5 g), liver weight (mean 6.4 ± 0.2 g), spleen weight (mean 0.4 ± 0.0 g), and splenocyte count (mean 154 ± 8 × 10^6^ cells) at 8 weeks of age (Supplementary Table 1).

### Fatty Acid Composition of Breastmilk (Mammary Gland) and Plasma of Dams

Breastmilk from dams fed the ARA+DHA maternal diet resulted in significantly higher DHA content (four times, *p* < 0.0001), total *n*−3 fatty acids, total n-6 fatty acids, total PUFA, and PUFA/SFA ratio when compared to the control diet *(p* < 0.05). Further, ARA+DHA maternal diet resulted in a significantly lower ARA/DHA ratio, total monounsaturated fatty acids (MUFA), α-linolenic acid (ALA), and C22:5*n*−6 content in breastmilk when compared to dams that were fed the control maternal diet (*p* < 0.05). However, supplementing the maternal diet with ARA + DHA did not alter ARA, *n*−6/*n*−3 ratio, and total SFA content of breastmilk total lipids (Table 2). Note, feeding ARA + DHA maternal diet to breastfeeding dams also resulted in significantly higher plasma total phospholipid content of DHA (1.5 ± 0.2 vs. 9 ± 0.1, *p* = 0.04) when compared to the maternal control diet (*p* = 0.04) without affecting the ARA content (Supplementary Table 2).

**Table 2:** Fatty acid composition of total lipids in breastmilk from the mammary gland of Brown Norway dams at the end of suckling period^a^.

**Fatty acid (g/100 g total fatty acids)**	**Control diet**	**ARA+DHA diet**	***P-*maternal**
	**(*n* = 4)**	**(*n* = 6)**	**diet**
14:0	1.4 ± 0.1	1.3 ± 0.0	0.27
16:0	20.8 ± 0.4	20.1 ± 0.5	0.36
18:0	10.0 ± 0.5	11.6 ± 0.8	0.16
20:0	0.1 ± 0.0	0.1 ± 0.0	0.88
24:0	0.1 ± 0.0	0.2 ± 0.0	0.29
16:1*n*−9	1.9 ± 0.2	1.5 ± 0.1	0.05
18:1*n*−9 oleic acid	43.0 ± 0.8	38.5 ± 0.9	0.008
24:1*n*−9	0.1 ± 0.0	0.1 ± 0.0	0.43
18:2*n*−6 LA	16.9 ± 0.4	19.1 ± 0.3	0.001
20:2*n*−6	0.4 ± 0.0	0.5 ± 0.0	0.03
20:3*n*−6	0.2 ± 0.0	0.3 ± 0.0	0.33
20:4*n*−6 ARA	1.6 ± 0.1	2.2 ± 0.3	0.12
22:4*n*−6	0.2 ± 0.0	0.2 ± 0.0	0.98
22:5*n*−6	0.07 ± 0.0	0.1 ± 0.0	<0.001
18:3*n*−3 ALA	1.7 ± 0.1	1.5 ± 0.1	0.02
20:5*n*−3 EPA	0.0 ± 0.0	0.0 ± 0.0	0.96
22:5*n*−3 DPA	0.1 ± 0.0	0.1 ± 0.0	0.66
22:6*n*−3 DHA	0.2 ± 0.0	0.8 ± 0.0	<0.0001
Total^b^ SFA	33.6 ± 0.6	35.0 ± 0.7	0.21
Total MUFA	44.9 ± 0.8	40.1 ± 0.9	0.008
Total PUFA	21.5 ± 0.6	24.9 ± 0.3	0.001
Total *n*−6	19.5 ± 0.5	22.4 ± 0.3	0.001
Total *n*−3	2.0 ± 0.1	2.5 ± 0.1	0.002
Ratio *n*−6/*n*−3	9.6 ± 0.1	9.1 ± 0.3	0.31
Ratio PUFA/SFA	0.6 ± 0.0	0.7 ± 0.0	0.015
Ratio ARA/DHA	9.0 ± 0.9	2.9 ± 0.27	0.001

a *Data is presented in mean ± standard error of the mean. ALA, α-linolenic acid; LA, linoleic acid; ARA, arachidonic acid; DHA, docosahexaenoic acid; EPA, eicosapentaenoic acid; DPA, docosapentaenoic acid; PUFA, polyunsaturated fatty acid; MUFA, monounsaturated fatty acid; SFA, saturated fatty acid*.

b *Some fatty acids that were traced were not presented as they were very small or not relevant, which may not add up to the total amount such as total SFA, PUFA, MUFA, n−6, and n−3*.

### Fatty Acid Composition of Total Phospholipids in Tissues From 8-Week-Old Offspring

**Figure 2:**
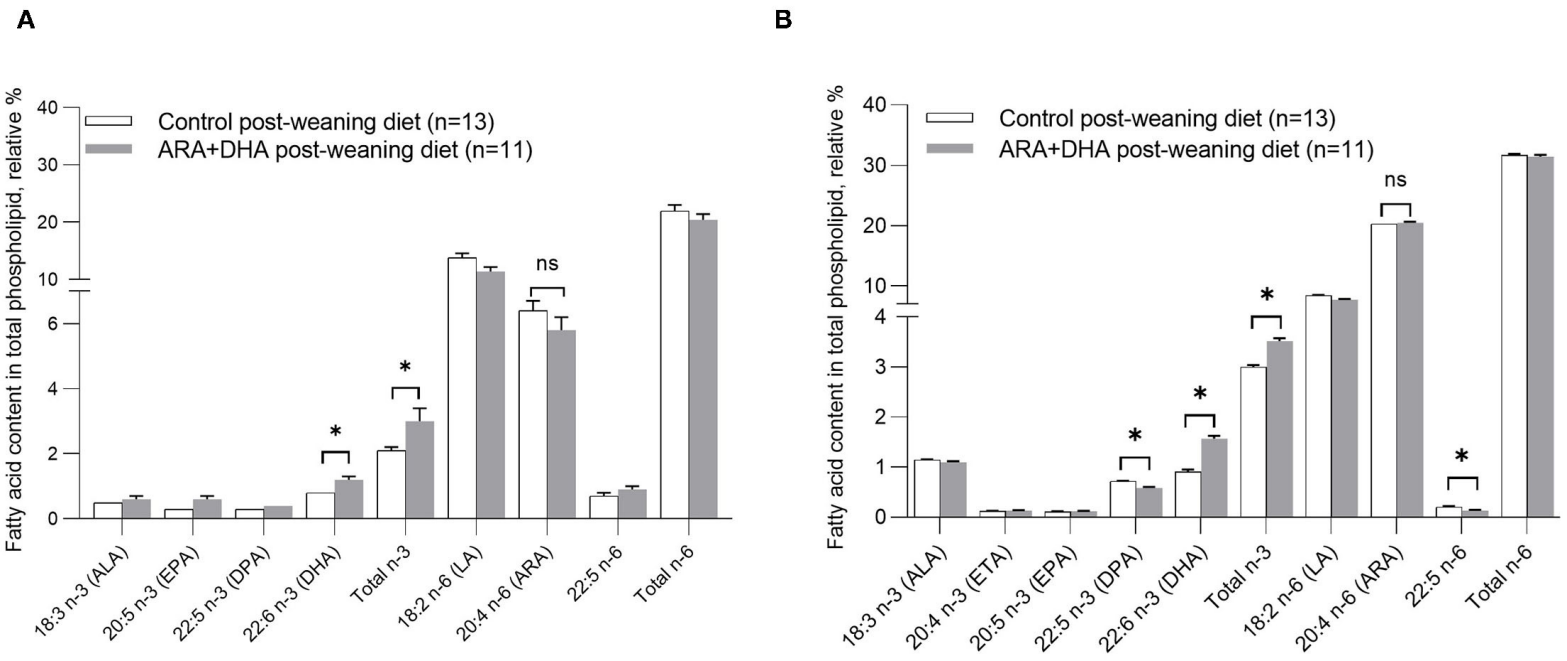
Effect of post-weaning diet on the relative % of fatty acid content in total phospholipids in **(A)** plasma and **(B)** splenocytes of 8-week-old Brown Norway offspring. All values are presented in mean ± SE. At 8 weeks, there was no significant effect of the suckling period diet (0–3 weeks) or the suckling diet × post-weaning diet interaction. Therefore, the suckling diet groups were combined, and the differences observed by significant post-weaning diet were calculated by unpaired Student *t-*test. *p < 0.05. ALA, α-linolenic acid; DPA, docosapentaenoic acid; EPA, eicosapentaenoic acid; DPA, docosapentaenoic acid; DHA, docosahexaenoic acid; LA, linoleic acid; ARA, arachidonic acid.

Regardless of the suckling diet, the ARA+DHA post-weaning diet resulted in 50% higher DHA (1.2 ± 0.1 vs. 8 ± 0.0, *p* = 0.02) in plasma phospholipids compared with the control post-weaning diet (Figure 2A). The suckling or post-weaning diets had no effects on ARA, LA, eicosatetraenoic acid (ETA), docosapentaenoic acid (DPA), ALA, total SFA, total MUFA, total PUFA, and total n-6 content of plasma phospholipids.

Splenocyte phospholipid fatty acid content of 8-week-old offspring was not significantly altered by the suckling diet. However, in offspring that were fed ARA + DHA post-weaning diet (regardless of suckling diet), the splenocyte phospholipid content of DHA (1.65 ± 0.04 vs. 0.85 ± 0.02; *p* < 0.001) and total *n*−3 fatty acid (3.6 ± 0.1 vs. 2.9 ± 0.0; *p* = 0.003) was higher compared to offspring that were fed the control post-weaning diet. Further, ARA + DHA post-weaning diet resulted in lower *n*−6/*n*−3 ratio (10.8 ± 0.1 vs. 8.7 ± 0.1; *p* < 0.001) compared to control weaning diet group. The ARA content was not significantly different across the diet groups (Figure 2B, Table 3).

**Table 3:** The effect of supplementing suckling diet and post-weaning diet on the total fatty acid composition in phospholipids of splenocytes of pups at 8 weeks^1^.

**Suckling diet**	**Control**	**Control**	**ARA + DHA**	**ARA + DHA**			
**Post-Weaning diet**	**Control**	**ARA + DHA**	**Control**	**ARA + DHA**			
**Fatty acid (g/100 g**	**Mean ± SEM**	**Mean ± SEM**	**Mean ± SEM**	**Mean ± SEM**			
**total fatty acids)**	**(*n* = 8)**	**(*n* = 7)**	**(*n* = 8)**	**(*n* = 9)**	***P*-SD^2^**	***P*-WD^3^**	***P*-SD × WD^4^**
14:0	0.3 ± 0.1	0.4 ± 0.1	0.4 ± 0.1	0.4 ± 0.1	0.99	0.77	0.86
16:0	24.3 ± 0.5	24.5 ± 0.3	24.5 ± 0.3	24.2 ± 0.2	0.87	0.72	0.37
18:0	20.7 ± 0.3	20.6 ± 0.3	21.2 ± 0.3	21.3 ± 0.5	0.32	0.83	0.05
20:0	0.3 ± 0.0	0.3 ± 0.0	0.3 ± 0.0	0.4 ± 0.0	0.11	0.43	0.14
24:0	0.6 ± 0.0	0.6 ± 0.0	0.7 ± 0.0	0.7 ± 0.0	0.15	0.57	0.36
16:1*n*−9	0.9 ± 0.0^ab^	1.0 ± 0.0^b^	0.9 ± 0.0^b^	0.8 ± 0.0^a^	0.29	0.81	0.001
18:1*n*−9 oleic acid	10.9 ± 0.1	10.6 ± 0.1	10.7 ± 0.1	10.5 ± 0.2	0.75	0.23	0.42
24:1*n*−9	3.6 ± 0.1	3.4 ± 0.1	3.6 ± 0.1	3.5 ± 0.1	0.98	0.11	0.41
18:2*n*−6 LA	8.3 ± 0.1^ab^	8.1 ± 0.2^b^	8.5 ± 0.2^b^	7.8 ± 0.3^a^	0.48	0.23	0.008
20:2*n*−6	1.0 ± 0.0	1.0 ± 0.0	1.0 ± 0.0	0.9 ± 0.0	0.49	0.18	0.14
20:3*n*−6	1.7 ± 0.0	1.7 ± 0.0	1.7 ± 0.0	1.7 ± 0.0	0.57	0.29	0.30
20:4*n*−6 ARA	20.5 ± 0.4	20.7 ± 0.1	19.8 ± 0.3	20.7 ± 0.3	0.25	0.14	0.16
22:4*n*−6	0.1 ± 0.0	0.1 ± 0.0	0.1 ± 0.0	0.1 ± 0.0	0.58	0.97	0.28
22:5*n*−6	0.2 ± 0.0	0.1 ± 0.0	0.2 ± 0.0	0.1 ± 0.0	0.17	<0.001	0.07
18:3*n*−3 ALA	1.1 ± 0.0	1.1 ± 0.0	1.1 ± 0.0	1.1 ± 0.0	0.47	0.41	0.69
20:4*n*−3 ETA	0.1 ± 0.0	0.1 ± 0.0	0.1 ± 0.0	0.1 ± 0.0	0.25	0.95	0.50
20:5*n*−3 EPA	0.1 ± 0.0	0.1 ± 0.0	0.1 ± 0.0	0.1 ± 0.0	0.80	0.46	0.14
22:5*n*−3 DPA	0.7 ± 0.0	0.6 ± 0.0	0.7 ± 0.0	0.6 ± 0.1	0.51	0.01	0.64
22:6*n*−3 DHA	0.8 ± 0.0	1.7 ± 0.0	0.8 ± 0.0	1.6 ± 0.1	0.84	<0.001	0.21
Total SFA	46.7 ± 0.5	46.8 ± 0.2	47.5 ± 0.5	47.3 ± 0.6	0.46	0.88	0.83
Total MUFA	18.4 ± 0.1	17.8 ± 0.0	18.2 ± 0.0	17.6 ± 0.1	0.46	0.10	0.54
Total PUFA	34.7 ± 0.5	35.3 ± 0.2	34.2 ± 0.4	34.9 ± 0.5	0.58	0.25	0.81
Total *n*−6	31.8 ± 0.4	31.6 ± 0.2	31.2 ± 0.4	31.3 ± 0.4	0.52	0.84	0.80
Total *n*−3	2.9 ± 0.1	3.6 ± 0.1	2.9 ± 0.0	3.6 ± 0.1	0.98	0.003	0.56
Ratio *n*−6/*n*−3	10.7 ± 0.2	8.7 ± 0.2	10.6 ± 0.1	8.6 ± 0.1	0.20	<0.001	0.41
Ratio PUFA/SFA	1.9 ± 0.0	2.0 ± 0.0	1.9 ± 0.0	2.0 ± 0.0	0.85	0.02	0.94

1 *Data is presented in mean ± SE of the mean. Means within a row without a common superscript letter are significantly different, p <0.05. ALA, α-linolenic acid; LA, linoleic acid; ARA, arachidonic acid; ETA, eicosatetraenoic acid; EPA, eicosapentaenoic acid; DPA, docosapentaenoic acid; DHA, docosahexaenoic acid; PUFA, polyunsaturated fatty acid; MUFA, monounsaturated fatty acid; SFA, saturated fatty acid; SD, suckling diet; WD, post-weaning diet*.

2 *Indicates p-value for the main effect of the SD in the mixed model*.

3 *Indicates p-value for the main effect of the WD in the mixed model*.

4 *Indicates interaction between maternal SD and WD of offspring*.

A significant suckling diet effect in the fatty acid content of MLN was observed in three instances with lower 14:0, higher LA, and a higher *n*−6/*n*−3 ratio for 8-week-old offspring from dams fed the ARA + DHA diet compared to control diet. There were no significant post-weaning diet effects observed in the MLN fatty acid composition (Supplementary Table 3). The composition of total n-3, ALA, EPA, and DHA showed no significant suckling diet effect or post-weaning diet effect (Supplementary Table 3). However, a suckling diet × post-weaning diet interaction was observed for the proportion of DPA in MLN phospholipids, with a lower proportion of DPA in offspring that received ARA+DHA during both suckling and post-weaning period compared to the group that received control for the suckling period and ARA+DHA for the post-weaning period (0.3 ± 0.1 vs. 0.5 ± 0.1, *p* = 0.01; Supplementary Table 3).

### *Ex vivo* Cytokine Response to Mitogens by Immune Cells

**Figure 3:**
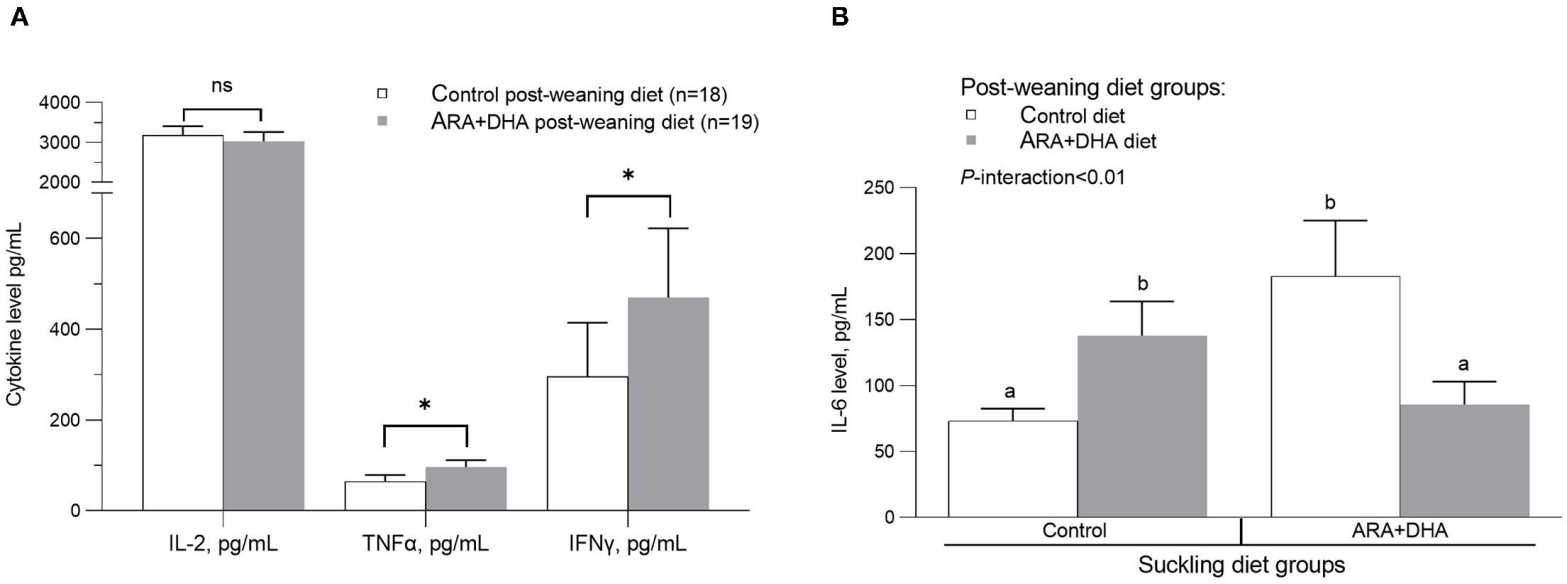
The effect of post-weaning diet on the *ex vivo* cytokine production after phorbol-myristate-acetate and ionomycin (PMAi) stimulation of splenocytes from 8 weeks offspring. Values are presented in mean ± SEM. **(A)** The post-weaning diet group comparison for IL-2 (*p* = 0.55), TNF-α (*p* = 0.02) and IFNγ (*p* = 0.04). The interaction of suckling diet and post-weaning diet main effects (*P*-interaction < 0.01) on *ex vivo* IL-6 production to PMAi stimulation is shown in **(B)**. The *p-*values for suckling diet and post-weaning diet are *p* = 0.81 and *p* = 0.92. *Post*-*hoc* differences are calculated using LSMEANS in the mixed model. The groups that do not share the same letter are significantly different. Note, Supplementary Table 4 reports the mean ± SE for the 4 diet groups as per the SD × WD interaction analysis. *, indicates statistical significance was assumed at *P* < 0.05.

Cytokine (IL1-β, TGF-β, TNF-α, IL-6, IL-10, and IFN-γ) production by splenocytes stimulated with LPS showed no suckling diet effect, post-weaning diet effect, or interactions (Supplementary Table 4). Production of IL-2 (as a surrogate marker of cell proliferation), TGF-β, IL-10, IL-4, and IL-13 with PMAi stimulation of splenocytes did not differ between groups. TNF-α and IFN-γ production by PMAi stimulated splenocytes was higher in offspring when they were weaned to the ARA+DHA diet compared with control offspring regardless of their suckling diet (30% higher, *p* = 0.02 and 60% higher, *p* = 0.04, respectively; Figure 3A). A significant interaction between the suckling diet and the post-weaning diet occurred in two instances. Specifically, PMAi stimulated splenocytes from offspring who received the control diet during suckling and post-weaning produced lower IL-6 while those who received ARA+DHA during the suckling period and then the control diet during the post-weaning period had the highest IL-6 production (73 ± 9 vs. 183 ± 42, *p* < 0.01, Figure 3B). With respect to IL-10 production, unstimulated splenocytes from offspring that received ARA+DHA during the suckling and post-weaning period had the lowest levels of IL-10 production compared with all other diet group offspring (*p* = 0.01, Supplementary Table 4).

In LPS stimulated MLN cells, there was a significant suckling diet × post-weaning diet interaction effect regarding IL-10 production where the offspring that received control diet during suckling and post-weaning period produced significantly higher IL-10 compared to the group that received ARA + DHA diet (either during suckling period or post-weaning period) and the group that received ARA+DHA throughout suckling and post-weaning (Supplementary Table 5). IL-2, TGF-β, IL-10, and TNF-α production by PMAi stimulated MLN cells did not differ among diet groups (Supplementary Table 5).

### Immune Cell Phenotype of Spleen

**Figure 4:**
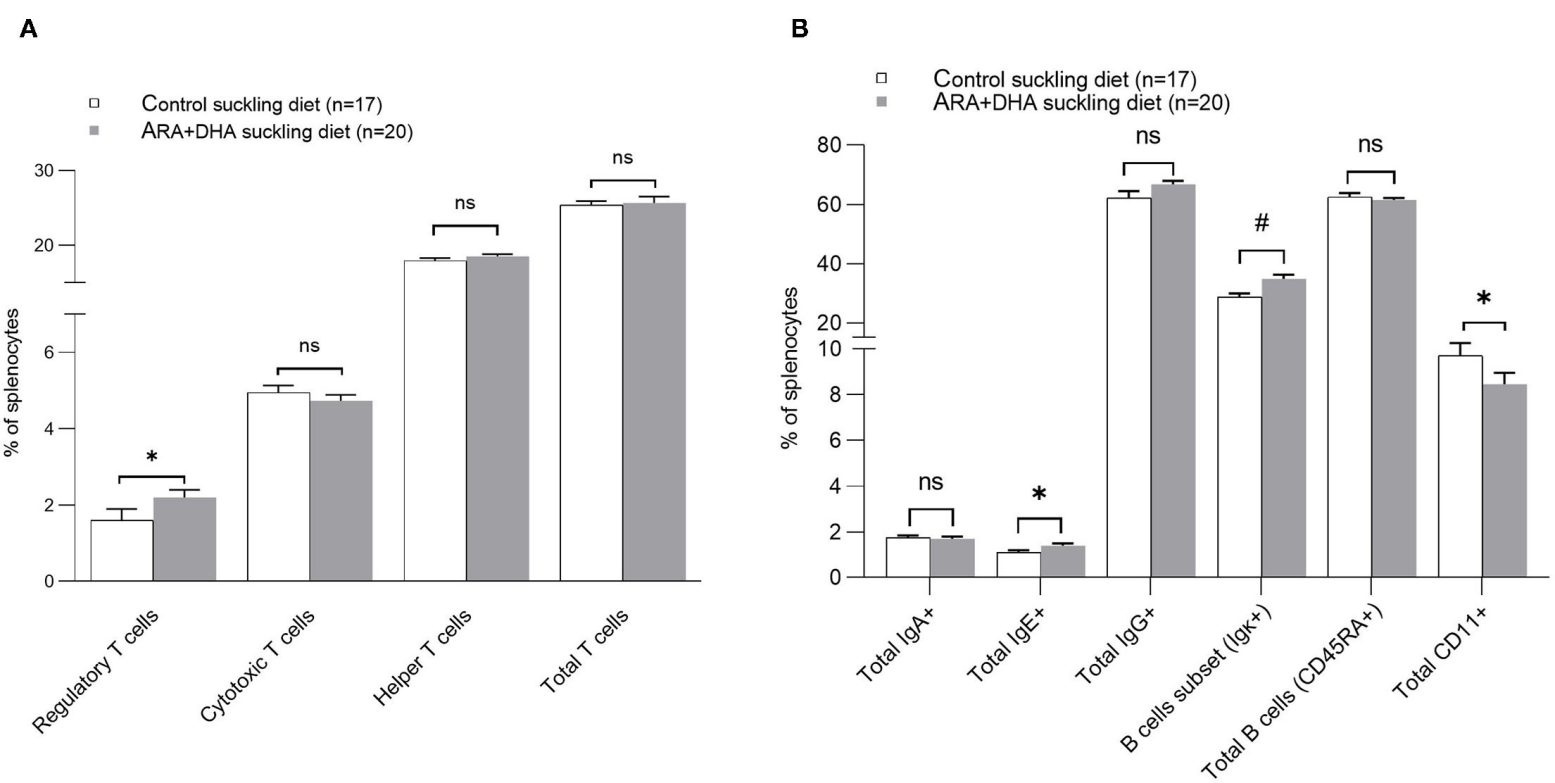
The effect of suckling diet on the immune cell phenotypes of spleen from 8 weeks offspring. The major proportion of T cells are shown **(A)** and other major immune cell phenotypes **(B)**. The values are shown in the mean ± SE for each suckling diet group. The *P*-value for the main effect of suckling diet [Regulatory T cells: *P* = 0.04, Helper T cell: *p* = 0.77, Cytotoxic T cells: *p* = 0.78, Total T cells: *p* = 0.83, IgA: *p* = 0.36, IgE: *p* = 0.02, IgG: *p* = 0.37, B cell subset: *p* = 0.001, Total B cells (CD45RA+): *p* = 0.35 and monocytes/macrophages (CD11+): *p* = 0.03] are also presented in Table 4. The significant suckling diet effect is shown by asterisk. The suckling diet and post-weaning diet interaction effect on the B cell subset are shown by the number sign (#), *P* interaction = 0.02. *, indicates statistical significance was assumed at *P* < 0.05.

Splenocyte proportion of total Th cells and cytotoxic T cells (CTL), dendritic cells (OX62+), antigen presenting cells (APC, OX6+), macrophage (CD68+), NK cells (CD3– CD161+), NKT cells (CD3+ CD161+), and total TCRαβ+ (αβ T cells) did not differ among groups. Note, the cell surface marker combinations and the associated cell populations are shown in Supplementary Table 6. However, significant effects of the suckling diet were observed. The splenocyte proportion of T regulatory cells (Treg, CD25+FOXp3+ on CD3+CD4+, *p* =0.04) was higher in the ARA+DHA suckling diet group compared to those who were suckled from control diet fed dams (Figure 4A). While there were no differences in total naïve leukocytes (CD45RA+), there was a higher proportion of activated B cells (OX12+, *p* < 0.001) in splenocytes in the ARA+DHA suckling diet group compared to the control suckling diet (36.6 ± 1.5 vs. 27 ± 0.9, *p* < 0.001). However, this was only observed when the offspring were weaned to control the post-weaning diet, as revealed by a significant suckling diet X post-weaning diet interaction (Table 4).

**Table 4:** The effect of supplementing suckling diet and post-weaning diet on the composition of immune cell types isolated from the spleen of 8-week-old Brown Norway offspring^1^.

**Suckling diet**	**Control**	**Control**	**ARA + DHA**	**ARA + DHA**			
**Post-Weaning diet**	**Control**	**ARA + DHA**	**Control**	**ARA + DHA**			
	**Mean ± SEM**	**Mean ± SEM**	**Mean ± SEM**	**Mean ± SEM**			
	**(*n* = 9)**	**(*n* = 8)**	**(*n* = 10)**	**(*n* = 10)**	***P*-SD^2^**	***P*-WD^3^**	***P*-SD × WD^4^**
**T cells subset marker**							
Total CD3+	25.2 ± 0.7	26.2 ± 0.6	25.0 ± 0.5	26.3 ± 1.0	0.83	0.28	0.49
Total TCRαβ+ (Tαβ cells)	23.4 ± 0.5	23.8 ± 0.6	24.0 ± 0.5	24.4 ± 0.8	0.99	0.35	0.91
Total CD4+	21.6 ± 0.4	21.7 ± 0.5	21.8 ± 0.6	22.5 ± 0.9	0.91	0.43	0.67
Total CD8+	8.4 ± 0.2	8.4 ± 0.1	8.2 ± 0.1	8.5 ± 0.1	0.84	0.30	0.47
FOXp3+ in CD3 + CD4 + CD25+ (Treg)	1.5 ± 0.2	1.7 ± 0.3	2.1 ± 0.2	2.2 ± 0.2	0.04	0.62	0.94
CD4+ in CD3+ (Th cells)	70.1 ± 1.3	71.4 ± 1.4	71.8 ± 1.0	72.4 ± 1.2	0.77	0.25	0.20
CD25+ in CD3+ CD4+ (activated Th cell)	12.0 ± 1.7	10.1 ± 0.4	11.6 ± 1.3	9.6 ± 0.4	0.99	0.13	0.65
CD8+ in CD3+ (CTL)	19.0 ± 0.6	20.0 ± 0.9	18.4 ± 0.5	18.3 ± 0.6	0.78	0.37	0.25
CD25+ in CD3+ CD8+ (activated CTL)	3.6 ± 0.2	3.6 ± 0.2	4.4 ± 0.5	3.7 ± 0.2	0.59	0.99	0.18
CD152+ in CD4+	3.2 ± 1.4	1.5 ± 0.3	2.8 ± 1.1	1.6 ± 0.4	0.99	0.46	0.91
CD152+ in CD8+	3.0 ± 0.8	1.5 ± 0.4	1.9 ± 0.7	1.0 ± 0.3	0.21	0.05	0.84
**B cell subset marker**							
Total CD45RA+	61.1 ± 1.1	63.9 ± 1.6	62.1 ± 0.6	60.8 ± 0.8	0.35	0.49	0.07
Total OX12+ (B cell subtype)	27.0 ± 0.9^c^	30.5 ± 1.5^bc^	36.6 ± 1.5^a^	33.0 ± 1.4^ab^	0.001	0.96	0.02
Total IgE+	1.1 ± 0.1	1.0 ± 0.0	1.4 ± 0.1	1.4 ± 0.1	0.02	0.90	0.48
**Innate immune cell subset marker**							
Total CD11+	10.1 ± 0.6	9.3 ± 0.4	8.6 ± 0.6	8.3 ± 0.4	0.03	0.35	0.68
Total OX6+ (APC–MHC-II)	50.8 ± 1.8	56.2 ± 1.7	56.1 ± 1.5	56.3 ± 1.3	0.31	0.09	0.12
Total OX62+ (dendritic cell)	4.9 ± 0.3	4.6 ± 0.2	4.8 ± 0.2	4.9 ± 0.4	0.39	0.39	0.33
Total CD68+ (macrophage)	0.9 ± 0.1	1.0 ± 0.2	1.2 ± 0.2	1.2 ± 0.3	0.07	0.88	0.37
Total CD284+ (TLR4)	1.0 ± 0.1	0.8 ± 0.1	1.2 ± 0.2	1.2 ± 0.1	0.03	0.49	0.30
Total CD86+ (CD28 ligand)	13.5 ± 0.8	14.2 ± 0.7	17.3 ± 0.8	17.6 ± 1.2	0.09	0.57	0.83
CD3- CD161+ (NK cell)	7.5 ± 0.4	7.1 ± 0.3	7.1 ± 0.2	7.5 ± 0.3	0.60	0.74	0.25

1 *Values are presented as mean ± SEM; values are reported as % of the total gated splenocytes. Means within a row without a common superscript letter are significantly different, p <0.05. SD, suckling diet; WD, post-weaning diet; APC, antigen presenting cell; Th, helper T; CTL, cytotoxic T lymphocytes; NKT, natural killer T lymphocytes; NK, natural killer; CTLA4, cytotoxic T lymphocyte associated protein-4; IL2Rα, Interleukin2 receptor α-chain; Tαβ, T cell with αβ receptor; TLR4, toll like receptor-4; TCR, T cell receptor; Treg, T regulatory cell; SD, suckling diet; WD, post-weaning diet; MFI, median florescence intensity*.

2 *Indicates p-value for the main effect of the SD in the mixed model*.

3 *Indicates p-value for the main effect of the WD in the mixed model*.

4 *Indicates interaction between maternal SD and WD of offspring*.

*Some immune cell markers had no significant effect of diets or sex therefore they were not included in the table. This includes total FOXp3+ cells, total CD27+ (memory marker) cells, CD4+ in CD27+ cells, CD8+ in CD27+ cells, Total CD28+ (T cell co-receptor) cells, CD28+ in CD8+ CD4- cells, CD28+ in CD8- CD4+ cells, CD4+ in TCR+ cells, CD8+ in TCR+ cells, total IgA+ cells, total IgG+ cells, total CD152+ (CTLA4+) cells, total CD25+ (IL2Rα) cells, total CD161+ cells, CD3+ CD161+ (NKT) cells, CD25+ in OX6+ (activated APC) cells, OX62+ in OX6+ cells, CD11+ in CD45RA+ cells, CD68+ in CD45RA+ cells, CD284+ in CD45RA+ cells, and CD68+ in CD284+ cells*.

Furthermore, at 8 weeks of age, there was a higher proportion of total IgE+ splenocytes (*p* = 0.02) and no changes in IgG+ or IgA+ cells in offspring suckled on the ARA+DHA diet compared to control suckling diet group offspring (Table 4).

The proportion of TLR-4+ (total CD284+, shown in Table 4) cells was significantly higher and the proportion of monocytes (total CD11+, shown in Figure 4B) was significantly lower in splenocytes from the ARA+DHA suckling diet group compared to control suckling diet fed offspring (*p* < 0.05). At 8 weeks, a significant post-weaning diet effect was observed in only one instance, showing a 50% lower proportion of CD8+CD4–CD152+ (CTLA4+ CTL) in total splenocytes from the ARA+DHA post-weaning compared to the control diet group (*p* = 0.05, Table 4). Note, the gating strategy for CD152+ CD8+ was conducted on CD4 cells to ensure only T cells were gated as T cell-specific antibodies could not be used due to limitations with the number of antibodies used in combination.

### Immune Cell Phenotype of MLN

The major immune cell populations in MLN, T cells (CD3+), αβ T cells (TCRαβ+), CTL (CD3 + CD8+), Th cells (CD3 + CD4+), APC (OX6+), B cells (OX12+), IgA+ cells, IgG+ cells, and IgE+ cells did not differ amongst groups. However, at 8 weeks, the proportion of naïve leukocyte marker (CD45RA+) was 10% lower (*p* =0.002) and a total of CD152+ cells in MLN were 4 times higher in offspring from the ARA+DHA suckling diet group compared with control group offspring (Table 5). The post-weaning diet supplementation of ARA+DHA resulted in a significantly lower proportion of CD3 + CD8 + CD25+ (17%, *p* = 0.05) and total CD8+ cells (12%, *p* = 0.04), with no differences amongst post-weaning groups in MLN proportions of total CD4+ cell (*p* = 0.15) or activated Th cell (CD3 + CD4 + CD25+, *p* = 0.44) compared to control post-weaning diet group (Table 5). Further, there was no significant interaction between suckling and post-weaning diets on different immune cell populations of MLN.

**Table 5:** The effect of supplementing suckling diet and post-weaning diet on the composition of immune cell types isolated from the mesenteric lymph nodes of 8-week-old Brown Norway offspring^a^.

**Suckling diet**	**Control**	**Control**	**ARA + DHA**	**ARA + DHA**			
**Post-Weaning diet**	**Control**	**ARA + DHA**	**Control**	**ARA + DHA**			
	**Mean ± SEM**	**Mean ± SEM**	**Mean ± SEM**	**Mean ± SEM**			
	**(*n* = 8)**	**(*n* = 6)**	**(*n* = 9)**	**(*n* = 9)**	***P*-SD^b^**	***P*-WD^c^**	***P*-SD × WD^d^**
**T cell subset markers**							
Total CD3+	54.3 ± 2.0	49.7 ± 2.3	54.8 ± 1.9	53.2 ± 0.8	0.39	0.25	0.41
Total TCRαβ+ (αβ T cells)	52.5 ± 2.0	51.7 ± 1.2	59.7 ± 2.8	58.9 ± 3.3	0.15	0.76	0.88
Total CD4+	50.8 ± 1.8	46.0 ± 1.6	52.1 ± 1.9	50.8 ± 1.1	0.30	0.15	0.24
Total CD8+	7.0 ± 0.5	6.1 ± 0.3	7.3 ± 0.4	6.5 ± 0.1	0.16	0.04	0.71
CD4+ in CD3+ (Th cell)	80.1 ± 1.5	79.6 ± 1.9	80.3 ± 1.1	79.8 ± 2.5	0.96	0.85	0.78
CD25+ in CD3+ CD4+ (activated Th cell)	7.7 ± 0.6	7.0 ± 0.5	6.6 ± 0.3	6.6 ± 0.3	0.18	0.44	0.30
CD8+ in CD3+ (CTL)	8.2 ± 0.8	7.7 ± 0.5	7.7 ± 0.3	7.5 ± 0.2	0.63	0.63	0.98
CD25+ in CD3+ CD8+ (activated CTL)	10.1 ± 0.9	9.0 ± 1.0	10.7 ± 1.6	8.3 ± 1.1	0.15	0.048	0.44
**B cell subset markers**							
Total CD45RA+	46.0 ± 1.4	47.5 ± 1.3	42.0 ± 1.5	40.7 ± 1.6	0.002	0.94	0.38
Total OX12+ (B cells subtype)	18.6 ± 3.2	19.6 ± 2.3	15.6 ± 2.8	13.7 ± 2.5	0.18	0.99	0.26
Total IgA+	4.0 ± 0.5	4.6 ± 1	3.9 ± 0.5	2.6 ± 0.3	0.11	0.36	0.15
Total IgG+	38.6 ± 3.6	44.2 ± 1.7	38.9 ± 1.7	39.7 ± 1.5	0.26	0.33	0.45
Total IgE+	4.9 ± 0.5	5.9 ± 1	3.7 ± 0.6	5.2 ± 0.9	0.30	0.10	0.86
**Innate immune cell subset markers**							
Total CD28+ (T cell co-receptor)	49.1 ± 1.4	46.2 ± 1.4	56.7 ± 3.9	52.3 ± 1.1	0.18	0.17	0.44
Total CD152+ (CTLA4)	0.1 ± 0.0	0.1 ± 0.0	0.6 ± 0.2	0.5 ± 0.2	0.03	0.93	0.53
Total OX6+ (APC–MHC-II)	20.5 ± 3.3	20.9 ± 2.7	14.2 ± 1.4	13.0 ± 1.4	0.16	0.67	0.35
Total CD27+	72.6 ± 4.2	72.6 ± 3.8	76.2 ± 3.2	79.0 ± 3.3	0.09	0.35	0.41

a *Values are presented as mean ± SEM; values are reported as % of the total gated immune cells. SD, suckling diet; WD, post-weaning diet; APC, antigen presenting cell; Th, helper T; CTL, cytotoxic T lymphocytes; CTLA4, cytotoxic T lymphocyte associated protein-4; IL2Rα, Interleukin2 receptor α-chain; Tαβ, T cell with αβ receptor; TLR4, toll like receptor-4; TCR, T cell receptor; SD, suckling diet; WD, post-weaning diet*.

b *Indicates p-value for the main effect of the SD in the mixed model*.

c *Indicates p-value for the main effect of the WD in the mixed model*.

d *Indicates interaction between maternal SD and WD of offspring*.

*Some immune cell markers had no significant effect on diets or sex, therefore they were not included in the table. This includes CD4+ in TCRαβ+ cells, CD8+ in TCRαβ+ cells, CD27+ in CD4+ TCR+ cells, CD27+ in CD8+ TCR+ cells, and total CD25+ (IL2Rα) cells*.

### Immune Cell Phenotype of PP

Peyer's patches of 8-week-old offspring showed that the proportion of major immune cell subsets, such as T cells (CD3+), Th cells (CD3 + CD4+), CTL (CD3+CD8+), APC (OX6+), and naive leukocytes (CD45RA+) did not differ amongst groups (Table 6). A significant suckling diet effect was observed in one instance with a 30% higher proportion of total CD4+ cells in offspring that received ARA+DHA diet during suckling compared to control suckling diet at the end of 8 weeks (*p* = 0.001). Next, a significant post-weaning diet effect revealed that the total IgG+ cells in 8 weeks old offspring that received ARA+DHA post-weaning diet were approximately 30% higher compared to control offspring (*p* = 0.004). Finally, a suckling diet × post-weaning diet interaction was observed for total IgE+ cells with a significantly lower proportion in offspring who received the ARA + DHA suckling diet and control post-weaning diet group compared to ARA+DHA suckling diet and ARA + DHA post-weaning diet (*p* = 0.04, Table 6).

**Table 6:** The effect of supplementing suckling diet and post-weaning diet on the composition of immune cell types isolated from the Peyer's patches of 8-week-old Brown Norway offspring^1^.

**Suckling diet**	**Control**	**Control**	**ARA + DHA**	**ARA + DHA**			
**Post-Weaning diet**	**Control**	**ARA + DHA**	**Control**	**ARA + DHA**			
	**Mean ± SEM**	**Mean ± SEM**	**Mean ± SEM**	**Mean ± SEM**			
	**(*n* = 8)**	**(*n* = 7)**	**(*n* = 9)**	**(*n* = 9)**	***P*-SD^2^**	***P*-WD^3^**	***P*-SD × WD^4^**
**T cell subset marker**							
Total CD3+ (T cells)	43.0 ± 4.9	38.4 ± 2.9	43.2 ± 3.4	39.3 ± 2.9	0.94	0.40	0.41
Total CD4+	20.1 ± 4.2	21.6 ± 4.7	27.9 ± 4.1	26.5 ± 4.1	0.001	0.87	0.38
Total CD8+	10.2 ± 1.5	9.2 ± 2.5	9.9 ± 1.5	8.6 ± 2.1	0.94	0.39	0.53
CD4+ in CD3+ (Th cells)	41.5 ± 8.7	47.3 ± 9.0	43.6 ± 6.7	44.1 ± 5.1	0.33	0.35	0.21
CD8+ in CD3+ (CTL)	18.6 ± 2.5	17.5 ± 3.6	18 ± 2.4	17.4 ± 2.8	0.63	0.44	0.64
CD25+ in CD3+CD4+ (activated Th cells)	20.5 ± 3.6	22.8 ± 5.3	23.6 ± 3.7	19.9 ± 3.4	0.39	0.74	0.08
CD25+ in CD3+ CD8+ (activated CTL)	53.4 ± 3.3	52.9 ± 4.7	47.2 ± 5.3	42.7 ± 5.1	0.40	0.54	0.68
**B cell subset marker**							
Total CD45RA+	52.1 ± 4.4	58.7 ± 3.5	50.4 ± 2.1	60.6 ± 2.5	0.56	0.14	0.39
Total IgA+	6.3 ± 1.1	3.5 ± 0.9	7.5 ± 1.0	5.0 ± 1.2	0.45	0.27	0.44
Total IgG+	57.9 ± 5.4	69.2 ± 4.7	46.7 ± 5.3	65.4 ± 3.1	0.13	0.004	0.45
Total IgE+	4.2 ± 1.1^a^	3.7 ± 1.2^a^	7.2 ± 0.7^b^	4.9 ± 1.0^a^	0.07	0.09	0.04
**Innate immune cell subset markers**							
Total CD25+ (IL2Rα)	11.6 ± 1.6	8.4 ± 2.0	14.4 ± 1.7	11.1 ± 2.4	0.65	0.06	0.67
Total OX62+	11.9 ± 2.1	17.8 ± 3.1	16.6 ± 2.4	15.8 ± 2.8	0.92	0.20	0.06
Total CD86+	16.4 ± 3.4	14.4 ± 1.9	19.4 ± 2.7	21.2 ± 3.1	0.28	0.68	0.83
Total OX6+ (APC–MHC-II)	64.5 ± 3.9	64.5 ± 4.0	53.5 ± 3.3	59.5 ± 4.9	0.46	0.43	0.32

1 *Values are presented as mean ± SEM; values are reported as % of the total gated immune cells. Means within a row without a common superscript letter are significantly different, p <0.05. SD, suckling diet; WD, post-weaning diet; APC, antigen presenting cell; Th, helper T; CTL, cytotoxic T lymphocytes; IL2Rα, Interleukin2 receptor α-chain*.

2 *Indicates P-value for the main effect of the SD in the mixed model*.

3 *Indicates P-value for the main effect of the WD in the mixed model*.

4 *Indicates interaction between maternal SD and WD of offspring*.

### Sex Differences in Offspring at 8 Weeks

All parameters described here for the 8-week-old offspring were first assessed for differences by sex. We found in the MLN that total PUFA phospholipid content was higher in males vs. females (21.5 ± 1.9 vs. 16.9 ± 1.9, *p* = 0.007), however, the composition of ARA or DHA did not differ. Additionally, a significant sex effect was observed in PP with CD8+ cells being significantly higher in PP of female offspring compared to male (11.6 ± 1.7 vs. 8.3 ± 1.0, *p* < 0.05). No other significant differences due to sex were observed for any growth parameters, immune cell *ex vivo* cytokine production, immune cell phenotype, or fatty acid phospholipid composition. Therefore, the data below was pooled for sexes, and it is described as per their diet groups only.

## Discussion

**Figure 5:**
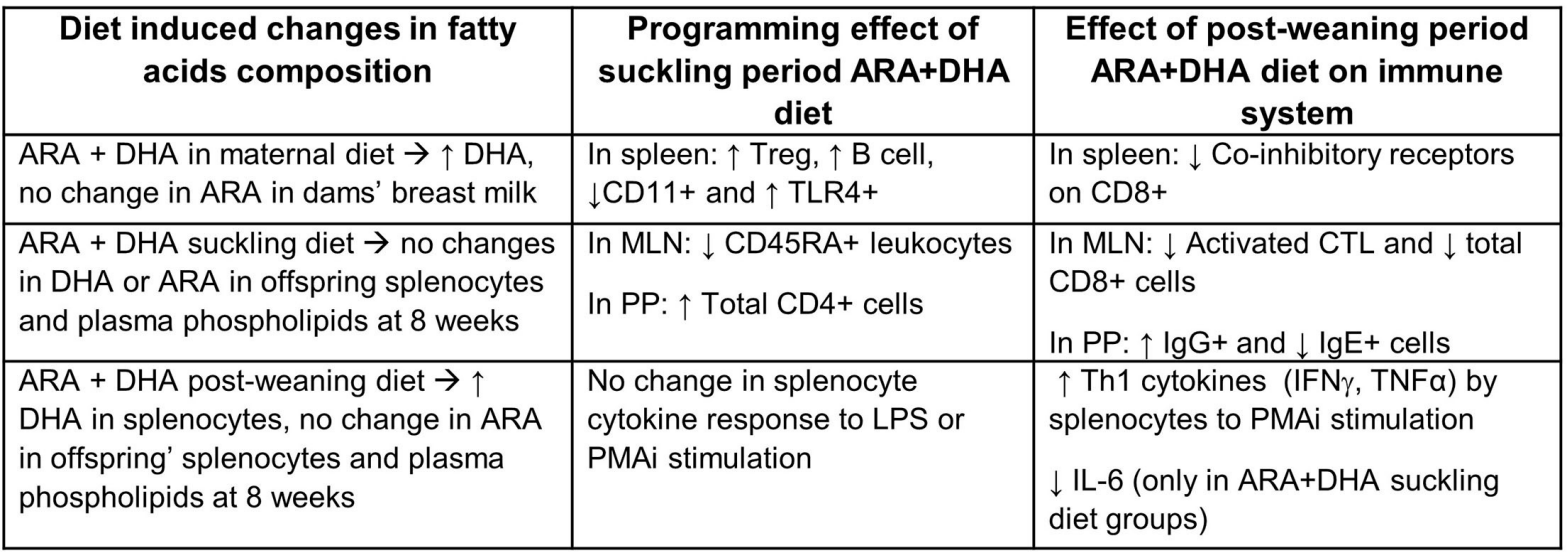
Summary of significant effects of supplementing ARA + DHA in infant diets on the immune system development of Brown Norway offspring. ↑, increase; ↓, decrease; ARA, arachidonic acid; DHA, docosahexaenoic acid; Treg, T regulatory cell, CTL, cytotoxic T cells; TLR4, toll-like-receptor 4; LPS, lipopolysaccharide; MLN, mesenteric lymph node; PP, Peyer's patches.

Using a Th2 bias Brown-Norway rodent model, we investigated the effect of supplementing the diet with ARA and DHA during critical immune development periods. The maternal diet supplementation of ARA+DHA resulted in higher DHA composition in the breastmilk total lipids and plasma phospholipids of dams. However, there was no change in the proportion of ARA. ARA+DHA supplementation through the suckling period (0–3 weeks) showed no lasting effects on the phospholipid composition of DHA or ARA in splenocytes or MLN in offspring at 8 weeks. Despite this, suckling period supplementation resulted in a programming effect on the types of immune cells in the spleen (Treg and B cell), MLN (naïve lymphocytes), and PP (IgG+ B cells), in that there were more mature immune cells observed at the end of the post-weaning period in offspring from dams fed ARA+DHA suckling diet. More importantly, the post-weaning period (3–8 weeks) ARA+DHA supplementation was associated with higher Th1 cytokines, such as TNF-α and IFN-γ, and lower IL-6 (which can be considered Th2 cytokine) after mitogen stimulation, indicating a shift from Th2 to Th1 immune response (Figure 5). Furthermore, differences due to weaning diet on immune cell types or proliferation (based on IL-2 production by splenocytes) involved in Th1 cytokine production were absent.

### Effect of Suckling Period and Post-weaning Period Supplementation on Fatty Acid Composition of 8-Week-Old Offspring

Offspring from ARA+DHA fed dams received, through breastmilk, significantly more DHA (from 0.2 to 0.8% of total fatty acid) and similar ARA (approximately 2% of total fatty acids). As expected, this effect of the 3-week suckling period diet supplementation on fatty acid composition was no longer present at the end of 8 weeks, where the fatty acid composition was influenced by the post-weaning diet. Consistent with other studies, growth parameters, such as body weight and length, were not different amongst dietary groups (12, 37). Offspring fed the ARA+DHA post-weaning diet, regardless of the suckling diet, had a higher DHA composition, without affecting the ARA in plasma and splenocytes phospholipids at 8 weeks. Although the increase in DHA breastmilk levels with dietary supplementation is dose dependent, ARA level in breastmilk is mainly dependent on maternal stores (31, 38). In the current study, dietary supplementation of ARA along with DHA was able to maintain ARA while increasing the breastmilk DHA composition in dams. Previous experiments from our lab with Sprague-Dawley rats have also reported similar changes at the end of the post-weaning period (13). Contrary to our hypothesis, at the current dietary concentration (0.5% DHA w/w of total fat), post-weaning diet supplementation did not appear to be sufficient to induce changes in the total phospholipid fatty acid composition of the immune cells from MLN. LCPUFA metabolism in different lymphoid tissue may vary depending on dominant immune cell type and function which may affect the tissue composition of fatty acids (39). Clinical trials have also reported tissue specific changes in fatty acid composition (40).

### Programming Effect of Suckling Period Supplementation on Immune Cell Phenotype in the Spleen, MLN, and PP

Dietary supplementation during the critical window of development in early infancy can result in changes, referred to as a programming effect, that can be observed later in life (41, 42). Studies have shown that *n*−3 supplementation can also have a programming effect using an animal model (12) and in clinical trials (16, 43, 44). Our data suggest that providing ARA and DHA during the suckling period had a programming effect on the maturation status of immune cell populations (summarized in Figure 5). The population of adaptive immune cells in the spleen increases during early infancy to match adult-levels as they mature. Specifically, splenocyte proportions of B cells, T cells, and NK cells increase during the suckling period (45) and additional immune cell maturity is achieved as food components are introduced during the post-weaning period (46, 47). Our findings in a Th2 bias Brown Norway rat are consistent with other LCPUFA supplementation studies showing higher B cells and T cell memory marker (CD27) in Sprague-Dawley rat offspring at the end of post-weaning (13). Furthermore, clinical trials have shown that 4 weeks of LCPUFA supplementation of formula during the suckling period in infants also resulted in higher adaptive cells such as T cells and CD4 + CD28+ cells in peripheral blood mononuclear cells (37). Next, we found that ARA+DHA suckling period supplemented offspring had a lower proportion of innate immune cell marker (CD11) that is generally expressed on granulocytes, dendritic cells, monocytes, and higher TLR-4+ splenocytes at the end of 8 weeks irrespective of the post-weaning diet. LPS, a bacterial component known to trigger antigen presenting cells, B cells, and macrophages can bind to TLR4 and induce activation of the innate immune response (48). However, no differences were observed in the *ex vivo* cytokine response to LPS with ARA+DHA supplementation at the end of post-weaning. This suggests that while suckling period supplementation (0–3 weeks) of ARA and DHA can promote higher adaptive immune cells, it is not associated with any changes in their ability to produce Th1 or Th2 response to *ex vivo* mitogen challenges after the post-weaning period.

Consistent with our findings in the spleen, suckling period ARA + DHA supplementation did not alter the proportion of the total T cells, B cells, NK cells, or APCs in the MLN at 8 weeks. However, CD45RA+ cells were significantly lower in the supplemented group offspring. As the lymphocytes circulate in the lymph node and encounter an antigen, it transitions from naïve to mature phenotype, which is marked by switching of CD45 isoform from CD45RA to CD45RO (49–51). Although we did not analyze the CD45RO cell surface marker in the current study, a decrease in CD45RA surface marker on lymphocytes is generally associated with an increase in CD45RO markers in circulating lymphocytes as the immune system matures (52). Hence, lower naïve lymphocytes in MLN observed in the ARA+DHA group offspring can be perceived as more mature lymphocytes. Furthermore, we found that immune cells of PP consisted of more CD4+ cells in ARA + DHA supplemented suckling diet group offspring regardless of the post-weaning diet at 8 weeks. The PP also showed a higher proportion of IgG+ cells and a lower proportion of IgE+ cells (only ARA+DHA suckling group) due to post-weaning period ARA + DHA supplementation with no differences in IgA+ cells or total B cells. Specialized pro-resolving mediators synthesized from DHA can enhance B cell differentiation and antibody production (53). However, DHA has also been shown to reduce IgE class-switching and production (54). Our data indicate that there were tissue specific changes in the phenotype of the immune cells, showing a more mature phenotype in the spleen, MLN, and PP. However, unlike other reports that showed changes in *ex vivo* cytokine production with stimulation, such as higher IL-10 responses by T cells (13), Th1 type cytokines to phytohemaglutinin (15, 55), and food antigens (56), we observed no functional changes in the splenocytes *ex vivo* cytokine response by adaptive immunity (PMAi stimulation) or innate immunity (LPS stimulation) for the suckling period ARA + DHA supplementation in 8-week-old Brown Norway offspring.

### Post-weaning Period ARA and DHA Supplementation, Regardless of the Suckling Diet, Is Associated With a Higher Th1 Cytokine Response

Production of higher Th1 cytokines can be an indicator of a reduction in Th2 response which is considered a more mature immune response in weaned infants (17). We have demonstrated for the first time in a Th2 biased model that ARA+DHA diet supplementation in the post-weaning period, irrespective of suckling diet, resulted in higher Th1 cytokines (IFN-γ and TNF-α) upon *ex vivo* lymphocyte stimulation with PMAi. An interaction of B7 molecule of APC with a co-stimulatory (CD28) and co-inhibitory (CD152 as known as CTLA-4) molecule of naïve T cell determines its overall effect (57). We showed splenocytes with lower co-inhibitory receptors (CD8 + CD152+) in ARA+DHA post-weaning offspring, with no differences in co-stimulatory marker (CD4 + CD28+ or CD8 + CD28+). This may also promote the Th1 cytokine response, as the CD152 marker has been reported to affect the T cell polarization (58). Our findings are consistent with those reported by a clinical study where DHA + EPA (fish oil) supplementation in Th2 biased allergy prone infants showing increased Th1 cytokines (IFN-γ and TNF-α) and reduced Th2 cytokine (IL-13) to PHA (mitogen stimulating T cells) (55). Furthermore, these immunomodulatory properties were shown to be protective of food allergies later in life (55).

The anti-inflammatory effects of n-3 LCPUFA on the immune system (due to increased Th2 cytokines, IL-4, IL-5, and IL-13, along with immunoregulatory IL-10 cytokine) are widely reported (59) especially with higher dietary intakes of EPA and/or DHA. It is hypothesized that ARA (and other n-6 LCPUFA) counters the anti-inflammatory effects of EPA and DHA, which may be responsible for reducing Th1 response by splenocytes in rodents (60). In addition, higher Th1 cytokines are also known to suppress Th2 cytokine production. However, the current study found no differences in key Th2 cytokines, namely, IL-4, IL-13, or TGF-β to PMAi due to ARA+DHA post-weaning diet supplementation. These findings once again demonstrate that a more mature immune response is associated with post-weaning period ARA+DHA supplementation where a higher Th1 cytokine was observed without any changes in Th2 cytokines or lymphocyte proliferation marker (IL-2 was used as an indirect measure of proliferation as it plays a vital role in lymphocyte proliferation). Lastly, we reported a lower IL-6 production with PMAi stimulation with ARA + DHA post-weaning diet compared to the controls group (when the suckling period was also supplemented). This is also consistent with other DHA supplementation studies in rodents (13, 61) and human feeding trials (62, 63). The anti-inflammatory effects of DHA have been hypothesized to be operating through the inhibition of NF-κB, which may mediate its beneficial role in various inflammatory diseases (64).

The current study has some limitations. Due to the small litter size of the Brown Norway dams, we had to conduct our animal experiments in 4 blocks. Statistical analysis was conducted to address random errors on the main effects due to differences in the block. However, it was not entirely possible to control the block effect for some parameters. Second, the *ex vivo* cytokine response to the mitogens was measured at one time point (72 h post-incubation) and does not allow us to distinguish early cytokine response from the later response. It is known that some cytokines, such as IFN-γ from macrophages, are produced early on (within 24 h) as compared to cytokines released by lymphocytes that take longer stimulation. We included male and female offspring in our study to determine if there were sex differences. However, we did not observe major sex differences, apart from differences in total PUFA composition of MLN and the proportion of CD8+ immune cells of PP. As it has not been studied before, our study may not have been appropriately powered or designed to assess sex effect in Th2 bias Brown Norway offspring. Therefore, the sex effect in neonatal immune system development needs to be further studied.

## Conclusion

Our findings suggest that providing more DHA during the suckling period through maternal diet ARA + DHA supplementation programmed the development of the Th2 biased immune system of Brown Norway offspring. We demonstrated that suckling period supplementation had a beneficial effect on the maturation status of the immune cell population of the spleen, MLN, and PP regardless of the post-weaning diet. However, these changes were not associated with any functional differences in the ability of mature immune cells from the spleen to respond to polyclonal mitogens at 8 weeks. Providing ARA+DHA in post-weaning diet results in a higher Th1 cytokine response by the splenocytes without affecting the Th2 cytokine response. Overall, the current study shows the importance of the immunomodulatory properties of LCPUFA, when provided during the suckling and post-weaning periods, resulting in a higher Th1 response by immune cells. This may be important for preventing allergies in infants with a genetic predisposition to develop allergic diseases.

## Data Availability Statement

The original contributions presented in the study are included in the article/Supplementary Material, further inquiries can be directed to the corresponding author/s.

## Ethics Statement

The animal study was reviewed and approved by University of Alberta Animal Ethics Committee.

## Author Contributions

CF, CR, DP, SG, and MN designed the study, conducted research and analyzed data, and performed the statistical analysis. DP wrote the manuscript. CF had primary responsibility for final content. All authors read and approved the final manuscript.

## Funding

The funding for this study was received from an NSERC discovery grant (NSERC RGPIN-2017-04746) to CF. DP has received scholarships from AGES ALES University of Alberta. DP has also received awards from GSS Government of Alberta and the 2020 Fisher Scientific Graduate Scholarship for scholarly achievements.

## Conflict of Interest

The authors declare that the research was conducted in the absence of any commercial or financial relationships that could be construed as a potential conflict of interest.

## Publisher's Note

All claims expressed in this article are solely those of the authors and do not necessarily represent those of their affiliated organizations, or those of the publisher, the editors and the reviewers. Any product that may be evaluated in this article, or claim that may be made by its manufacturer, is not guaranteed or endorsed by the publisher.

## Supplementary Material

Supplementary Figure 1 **(A)** Representative plots of gating strategy used to identify subtypes of T cells, CD45RA+ cells, and CD11+ cells based on cell surface marker on splenocytes from 8-week-old Brown Norway rat offspring. The forward-scatter area (FSC-A) vs. side-scatter area (SSC-A) plot was used to draw the size gate to delimitate the lymphocytes region followed by an FSC-A vs. forward-scatter height (FSA-H) for doublet exclusions (single cells). Gates were drawn for single fluorescence positive cells such as CD3+ (total T cells), CD11+ (macrophages), CD45RA+ (total B cells). Further, 2-dimension CD8 vs. CD4 graph was plotted on CD3+ T cells to identify CD8 + CD4- (cytotoxic T cells) and CD8-CD4+ (helper T cells) population. Subsequently, single stain CD25 was plotted on CTL and Th cells to identify CD25+ on CD8 + CD3+ (activated CTL) and CD25+ on CD4 + CD3+ (activated Th) cells as percent of their parent generation. **(B)** Representative plots of gating strategy used to identify T regulatory cells based on cell surface marker (CD3, CD4, and CD25) and intracellular marker (FoxheadP3, FoxP3) on splenocytes from 8-week-old Brown Norway rat offspring. The FSC-A vs. SSC-A plot was used to draw the size gate to delimitate the lymphocytes region followed by an FSC-A vs. FSA-H for doublet exclusions (single cells). Gates were drawn for single fluorescence CD3+ cells followed by CD4+ cells. Then, 2-dimension FoxP3 vs. CD25 graph was plotted to identify FoxP3 + CD25+ on CD4 + CD3+ cells (regulatory T cells). Numbers for the gated cell population represent the percent of positive cells for the stated marker in the parent population. Cell marker combination and the associated cell population identified are shown in Supplementary Table 1.

Supplementary Table 1 Anthropometric measurements of 8-week offspring based on suckling diet and post-weaning diet^1^. ^1^Data is presented in mean ± standard error of mean. SD, suckling diet; WD, post-weaning diet ARA; arachidonic acid; DHA, docosahexaenoic acid. ^2^Indicates *P*-value for the main effect of the SD in mixed model. ^3^Indicates *P*-value for the main effect of the WD in mixed model. ^4^Indicates interaction between maternal SD and offspring WD.

Supplementary Table 2 Total fatty acid composition of plasma phospholipids from dams at the end of suckling period^1^. ^1^Data is presented in mean ± standard error of mean. ALA, α-linolenic acid; LA, linoleic acid; ARA, arachidonic acid; DPA, docosapentaenoic acid; EPA, eicosapentaenoic acid; DHA, docosahexaenoic acid; PUFA, polyunsaturated fatty acid; MUFA, monounsaturated fatty acid; SFA, saturated fatty acid; SD, suckling diet. Outliers were removed from the analysis resulting in n value less than total used for the experiment. ^2^Some fatty acids that were traced were not presented as they were very small or not relevant, which may not add-up to total amount such as total SFA, PUFA, MUFA, *n*−6 and *n*−3.

Supplementary Table 3 The effect of supplementing suckling diet and post-weaning diet on the total fatty acid composition in phospholipids of immune cells isolated from mesenteric lymph nodes of offspring at 8 weeks^1^. ^1^Data is presented in mean ± standard error of mean. Means within a row without a common superscript letter are significantly different, *P* < 0.05. ALA, α-linolenic acid; LA, linoleic acid; ARA, arachidonic acid; ETA, eicosatetraenoic acid; EPA, eicosapentaenoic acid; DPA, docosapentaenoic acid; DHA, docosahexaenoic acid; PUFA, polyunsaturated fatty acid; MUFA, monounsaturated fatty acid; SFA, saturated fatty acid; SD, suckling diet; WD, post-weaning diet. Outliers were removed from the analysis resulting in *n* value less than total used for the experiment. ^2^Indicates *P*-value for the main effect of the SD in mixed model. ^3^Indicates *P*-value for the main effect of the WD in mixed model. ^4^Indicates interaction between maternal SD and offspring WD.

Supplementary Table 4 The effect of supplementing suckling diet and post-weaning diet on *ex vivo* cytokine production by splenocytes upon stimulation with mitogens^1^. ^1^Data is presented in mean ± standard error of mean. Means within a row without a common superscript letter are significantly different, *P* < 0.05. SD, suckling diet; WD, post-weaning diet; ARA, arachidonic acid; DHA, docosahexaenoic acid; PMAi, phorbol-myristate-acetate and ionomycin; IL, interleukin; TGFβ, transforming growth factor-β; TNFα, tumor necrosis factor-α; IFNγ, interferon-γ; SEM, standard error of mean; SD, suckling diet; WD, post-weaning diet. ^2^Indicates *P*-value for the main effect of the SD in mixed model. ^3^Indicates *P*-value for the main effect of the WD in mixed model. ^4^Indicates interaction between maternal SD and offspring WD.

Supplementary Table 5 The effect of supplementing suckling diet and post-weaning diet on *ex vivo* cytokine production by mesenteric lymph node cells after stimulation with mitogens^1^. ^1^Data is presented in mean ± standard error of mean. Means within a row without a common superscript letter are significantly different, *P* < 0.05. ARA, arachidonic acid; DHA, docosahexaenoic acid; SEM, standard error of mean; SD, suckling diet; WD, post-weaning diet; PMAi, phorbol-myristate-acetate and ionomycin; IL, interleukin; TGFβ, transforming growth factor-β; TNFα, tumor necrosis factor-α. ^2^Indicates *P*-value for the main effect of the SD in mixed model. ^3^Indicates *P*-value for the main effect of the WD in mixed model. ^4^Indicates interaction between maternal SD and offspring WD.

Supplementary Table 6 Description of immune cells phenotype used to identify immune cell subpopulations from lymphoid tissues collected from 8-week-old Brown Norway rat offspring^1^. MLN, mesenteric lymph nodes; PP, peyer's patches; CTL Cytotoxic T cells; αβ-TCR, αβ-T cell receptors; Th, Helper T cells; Treg, regulatory T cells; APC, antigen presenting cells; CTLA-4, Cytotoxic T-Lymphocyte Associated Protein 4; TLR4-4, toll like receptor-4; DC, dendritic cells. ^1^The gating strategy for main cell types has been reported in Supplementary Figure 1.
