# Supplementary figures and images for: Long Chain Polyunsaturated Fatty Acids Docosahexaenoic Acid and Arachidonic Acid Supplementation in the Suckling and the Post-weaning Diet Influences the Immune System Development of T Helper Type-2 Bias Brown Norway Rat Offspring
Source: Front Nutr. 2021 Nov 1;8:769293. doi: 10.3389/fnut.2021.769293 (PMC8592062; doi:10.3389/fnut.2021.769293)

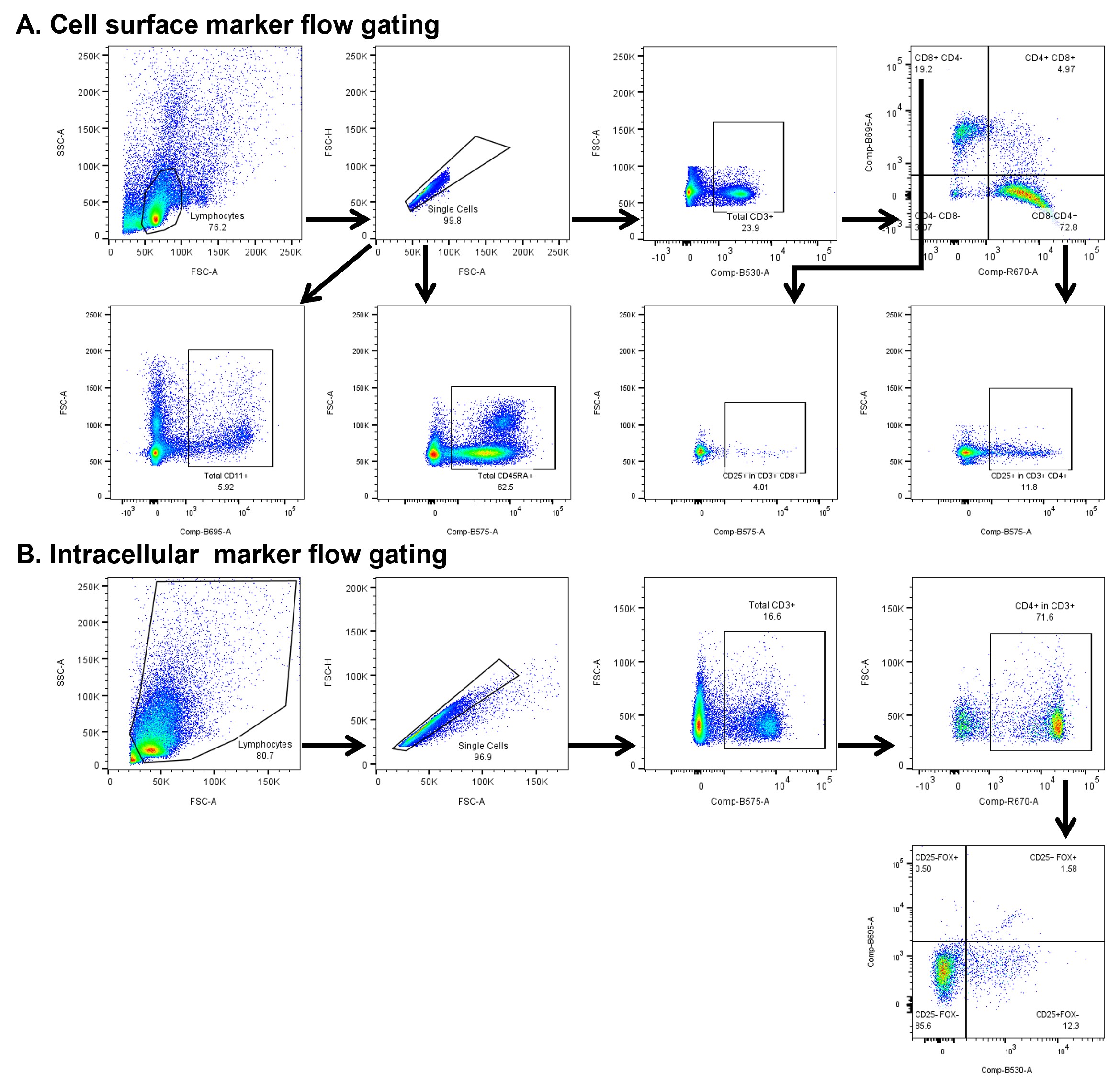

Supplement: Supplementary Figure 1 — (A) Representative plots of gating strategy used to identify subtypes of T cells, CD45RA+ cells, and CD11+ cells based on cell surface marker on splenocytes from 8-week-old Brown Norway rat offspring. The forward-scatter area (FSC-A) vs. side-scatter area (SSC-A) plot was used to draw the size gate to delimitate the lymphocytes region followed by an FSC-A vs. forward-scatter height (FSA-H) for doublet exclusions (single cells). Gates were drawn for single fluorescence positive cells such as CD3+ (total T cells), CD11+ (macrophages), CD45RA+ (total B cells). Further, 2-dimension CD8 vs. CD4 graph was plotted on CD3+ T cells to identify CD8 + CD4- (cytotoxic T cells) and CD8-CD4+ (helper T cells) population. Subsequently, single stain CD25 was plotted on CTL and Th cells to identify CD25+ on CD8 + CD3+ (activated CTL) and CD25+ on CD4 + CD3+ (activated Th) cells as percent of their parent generation. (B) Representative plots of gating strategy used to identify T regulatory cells based on cell surface marker (CD3, CD4, and CD25) and intracellular marker (FoxheadP3, FoxP3) on splenocytes from 8-week-old Brown Norway rat offspring. The FSC-A vs. SSC-A plot was used to draw the size gate to delimitate the lymphocytes region followed by an FSC-A vs. FSA-H for doublet exclusions (single cells). Gates were drawn for single fluorescence CD3+ cells followed by CD4+ cells. Then, 2-dimension FoxP3 vs. CD25 graph was plotted to identify FoxP3 + CD25+ on CD4 + CD3+ cells (regulatory T cells). Numbers for the gated cell population represent the percent of positive cells for the stated marker in the parent population. Cell marker combination and the associated cell population identified are shown in Supplementary Table 1. [file Image_1.jpeg]
