# Supplementary material for: Long Chain Polyunsaturated Fatty Acids Docosahexaenoic Acid and Arachidonic Acid Supplementation in the Suckling and the Post-weaning Diet Influences the Immune System Development of T Helper Type-2 Bias Brown Norway Rat Offspring
Source: Front Nutr. 2021 Nov 1;8:769293. doi: 10.3389/fnut.2021.769293 (PMC8592062; doi:10.3389/fnut.2021.769293)
Supplement: Supplementary Table 1 — Anthropometric measurements of 8-week offspring based on suckling diet and post-weaning diet1. 1Data is presented in mean ± standard error of mean. SD, suckling diet; WD, post-weaning diet ARA; arachidonic acid; DHA, docosahexaenoic acid. 2Indicates P-value for the main effect of the SD in mixed model. 3Indicates P-value for the main effect of the WD in mixed model. 4Indicates interaction between maternal SD and offspring WD. [file Data_Sheet_1.docx]

Supplementary Table 1. Anthropometric measurements of 8-week offspring based on suckling diet and post-weaning diet ^1^

| Suckling diet | Control | Control | ARA+DHA | ARA+DHA |  |  |  |
| --- | --- | --- | --- | --- | --- | --- | --- |
| Post-weaning diet | Control | ARA+DHA | Control | ARA+DHA | *P*-SD^2^ | *P*-WD^3^ | *P*-SD × WD^4^ |
|  | Mean ± SEM (n=9) | Mean ± SEM (n=8) | Mean ± SEM (n=10) | Mean ± SEM (n=10) |  |  |  |
| Body weight (g) | 153±10 | 149±12 | 158±8 | 164±9 | 0.23 | 0.69 | 0.79 |
| Animal length (cm) | 18.0±0.3 | 17.7±0.5 | 18.2±0.3 | 18.6±0.5 | 0.53 | 0.95 | 0.33 |
| Liver weight (g) | 6.3±0.4 | 6.1±0.4 | 6.4±0.4 | 6.6±0.3 | 0.17 | 0.87 | 0.41 |
| Spleen weight (g) | 0.4±0.0 | 0.4±0.0 | 0.4±0.0 | 0.4±0.0 | 0.22 | 0.86 | 0.27 |
| Total splenocytes (x10^6^ cells) | 152±17 | 134±14 | 175±17 | 151±18 | 0.38 | 0.35 | 0.72 |

^1^ Data is presented in mean ± standard error of mean. Abbreviations: SD, suckling diet; WD, post-weaning diet ARA; arachidonic acid; DHA, docosahexaenoic acid,

^2^ Indicates P-value for the main effect of the SD in mixed model.

^3^ Indicates P-value for the main effect of the WD in mixed model.

^4^ Indicates interaction between maternal SD and offspring WD.

Supplementary Table 2**.** Total fatty acid composition of plasma phospholipids from dams at the end of suckling period ^1^

| Fatty acid (g/100g total fatty acids) | Control diet (n=4) | ARA+DHA diet (n=6) | *P*-SD |
| --- | --- | --- | --- |
| 14:0 | 2.9±0.5 | 3.6±0.3 | 0.19 |
| 16:0 | 24.9±1.1 | 25.4±0.5 | 0.66 |
| 18:0 | 36.1±1.1 | 34.5±0.7 | 0.22 |
| 20:0 | 0.9±0.3 | 0.9±0.2 | 0.91 |
| 24:0 | 0.9±0.1 | 0.9±0.0 | 0.54 |
| 16:1n-9 | 0.6±0.2 | 0.3±0.1 | 0.13 |
| 18:1n-9 Oleic acid | 6.3±0.3 | 5.8±0.2 | 0.14 |
| 24:1n-9 | 0.9±0.2 | 0.8±0.1 | 0.94 |
| 18:2n-6 LA | 13.7±0.8 | 13.2±0.7 | 0.67 |
| 20:2n-6 | 0.5±0.1 | 0.7±0.1 | 0.35 |
| 20:3n-6 | 0.7±0.1 | 0.8±0.2 | 0.80 |
| 20:4n-6 ARA | 6.5±0.6 | 7.2±0.6 | 0.44 |
| 22:5n-6 | 0.6±0.1 | 0.7±0.1 | 0.40 |
| 18:3n-3 ALA | 0.9±0.2 | 0.7±0.1 | 0.55 |
| 20:5n-3 EPA | 0.4±0.1 | 0.4±0.1 | 0.93 |
| 22:5n-3 DPA | 0.5±0.1 | 0.5±0.1 | 0.88 |
| 22:6n-3 DHA | 0.9±0.1 | 1.5±0.2 | **0.04** |
| Total^2^ SFA | 66.4±1.1 | 66.2±0.7 | 0.88 |
| Total MUFA | 9.0±0.4 | 8.1±0.2 | **0.045** |
| Total PUFA | 24.6±0.8 | 25.7±0.6 | 0.29 |
| Total n-6 | 21.4±1.1 | 21.9±0.8 | 0.71 |
| Total n-3 | 2.6±0.4 | 3.1±0.2 | 0.29 |
| Ratio n-6/n-3 | 9.1±1.6 | 7.4±0.7 | 0.31 |

^1^ Data is presented in mean ± standard error of mean. Abbreviations: ALA, α-linolenic acid; LA, linoleic acid; ARA, arachidonic acid; DPA, docosapentaenoic acid; EPA, eicosapentaenoic acid; DHA, docosahexaenoic acid; PUFA, polyunsaturated fatty acid; MUFA, monounsaturated fatty acid; SFA, saturated fatty acid; SD, suckling diet. Outliers were removed from the analysis resulting in n value less than total used for the experiment.

^2^ Some fatty acids that were traced were not presented as they were very small or not relevant, which may not add-up to total amount such as total SFA, PUFA, MUFA, n-6 and n-3.

Supplementary Table 3. The effect of supplementing suckling diet and post-weaning diet on the total fatty acid composition in phospholipids of immune cells isolated from mesenteric lymph nodes of offspring at 8 weeks ^1^

| Suckling diet | Control | Control | ARA+DHA | ARA+DHA |  |  |  |
| --- | --- | --- | --- | --- | --- | --- | --- |
| Post-weaning diet | Control | ARA+DHA | Control | ARA+DHA | *P*-SD^2^ | *P*-WD^3^ | *P*-SD × WD^4^ |
| Fatty acid (g/100g total fatty acids) | Mean ± SEM (n=7) | Mean ± SEM (n=3) | Mean ± SEM (n=6) | Mean ± SEM (n=7) |  |  |  |
| 14:0 | 1.2±0.2 | 1.2±0.3 | 0.9±0.2 | 1.0±0.2 | 0.03 | 0.38 | 0.99 |
| 16:0 | 32.7±2.1 | 34.7±1.3 | 27.8±2.0 | 26.2±2.3 | 0.12 | 0.86 | 0.36 |
| 18:0 | 23.4±1.7 | 29.3±4.8 | 25.4±3.5 | 32.8±5.1 | 0.58 | 0.15 | 0.85 |
| 20:0 | 0.9±0.2 | 0.8±0.0 | 0.9±0.1 | 0.8±0.2 | 0.35 | 0.78 | 0.60 |
| 24:0 | 0.5±0.0 | 0.3±0.0 | 0.5±0.0 | 0.4±0.1 | 0.93 | 0.17 | 0.60 |
| 16:1n-9 | 1.0±0.0 | 0.8±0.0 | 1.2±0.1 | 0.8±0.0 | 0.52 | 0.10 | 0.38 |
| 18:1n-9 Oleic acid | 16.0±3.3 | 8.6±1.0 | 14.8±2.9 | 15.2±3.8 | 0.47 | 0.28 | 0.39 |
| 24:1n-9 | 0.7±0.1 | 1.1±0.3 | 0.9±0.2 | 0.5±0.1 | 0.15 | 0.79 | 0.45 |
| 18:2n-6 LA | 7.5±1.2 | 5.5±1.2 | 10.3±1.7 | 9.6±1.9 | 0.01 | 0.26 | 0.58 |
| 20:2n-6 | 0.7±0.1 | 0.9±0.1 | 0.9±0.1 | 0.7±0.1 | 0.53 | 0.90 | 0.08 |
| 20:3n-6 | 0.7±0.1 | 0.8±0.1 | 0.8±0.1 | 0.6±0.1 | 0.72 | 0.55 | 0.22 |
| 20:4n-6 ARA | 6.3±1.0 | 7.8±2.2 | 7.7±1.3 | 4.9±1.2 | 0.74 | 0.48 | 0.18 |
| 22:5n-6 | 1.3±0.3 | 1.1±0.2 | 1.1±0.2 | 1.0±0.3 | 0.54 | 0.35 | 0.97 |
| 18:3n-3 ALA | 1.3±0.1 | 1.2±0.2 | 1.2±0.1 | 1.1±0.1 | 0.68 | 0.42 | 0.83 |
| 20:5n-3 EPA | 0.6±0.2 | 0.5±0.1 | 0.6±0.2 | 0.3±0.1 | 0.28 | 0.58 | 0.89 |
| 22:5n-3 DPA | 0.4±0.1 ^ab^ | 0.5±0.1 ^b^ | 0.5±0.1 ^ab^ | 0.3±0.1 ^a^ | 0.03 | 0.95 | 0.01 |
| 22:6n-3 DHA | 0.8±0.2 | 1.0±0.1 | 0.7±0.1 | 0.6±0.2 | 0.28 | 0.87 | 0.31 |
| Total SFA | 60.3±4.1 | 67.7±5.4 | 56.7±2.4 | 62.1±4.7 | 0.52 | 0.27 | 0.88 |
| Total MUFA | 19.2±3.3 | 12.1±1.7 | 18.5±2.8 | 18.0±4.0 | 0.76 | 0.35 | 0.70 |
| Total PUFA | 19.9±1.9 | 20.6±2.7 | 24.0±1.6 | 19.4±2.1 | 0.17 | 0.21 | 0.33 |
| Total n-6 | 16.8±1.8 | 17.5±2.6 | 21.0±1.5 | 17.0±2.2 | 0.07 | 0.30 | 0.23 |
| Total n-3 | 3.1±0.5 | 3.1±0.3 | 3.0±0.4 | 2.4±0.2 | 0.23 | 0.64 | 0.75 |
| Ratio n-6/n-3 | 6.2±1.3 | 5.5±0.7 | 7.7±1.1 | 7.9±1.4 | 0.04 | 0.67 | 0.91 |
| Ratio ARA/DHA | 11.4±3.8 | 8.6±2.1 | 13.3±3.0 | 10.0±2.1 | 0.64 | 0.39 | 0.95 |

^1^ Data is presented in mean ± standard error of mean. Means within a row without a common superscript letter are significantly different, *P* < 0.05. Abbreviation; ALA, α-linolenic acid; LA, linoleic acid; ARA, arachidonic acid; ETA, eicosatetraenoic acid; EPA, eicosapentaenoic acid; DPA, docosapentaenoic acid; DHA, docosahexaenoic acid; PUFA, polyunsaturated fatty acid; MUFA, monounsaturated fatty acid; SFA, saturated fatty acid; SD, suckling diet; WD, post-weaning diet. Outliers were removed from the analysis resulting in n value less than total used for the experiment.

^2^ Indicates P-value for the main effect of the SD in mixed model.

^3^ Indicates P-value for the main effect of the WD in mixed model.

^4^ Indicates interaction between maternal SD and offspring WD.

Supplementary Table 4. The effect of supplementing suckling diet and post-weaning diet on *ex-vivo* cytokine production by splenocytes upon stimulation with mitogens ^1^

| Suckling diet | Control | Control | | ARA+DHA | ARA+DHA |  | | |
| --- | --- | --- | --- | --- | --- | --- | --- | --- |
| Post-weaning diet | Control | | ARA+DHA | Control | ARA+DHA | *P*-SD^2^ | *P*-WD^3^ | *P*-SD × WD^4^ |
|  | Mean ± SEM (n=9) | | Mean ± SEM (n=8) | Mean ± SEM (n=10) | Mean ± SEM (n=10) |  |  |  |
| **Lipopolysaccharide (pg/ml)** | | |  |  |  |  |  |  |
| IL-1β | 38±4 | | 27±2 | 28±3 | 27±2 | 0.77 | 0.06 | 0.08 |
| TGF-β | 314±19 | | 321±17 | 322±13 | 311±25 | 0.65 | 0.86 | 0.57 |
| TNF-α | 89±17 | | 80±18 | 98±20 | 113±24 | 0.60 | 0.89 | 0.83 |
| IL-6 | 419±41 | | 499±48 | 449±56 | 414±51 | 0.59 | 0.59 | 0.09 |
| IL-10 | 249±19 | | 258±35 | 239±30 | 240±14 | 0.70 | 0.84 | 0.86 |
| IFN-γ | 343±78 | | 757±227 | 603±158 | 319±62 | 1.00 | 0.97 | 0.06 |
| **PMAi (pg/ml)** | | |  |  |  |  |  |  |
| IL-2 | 3063±397 | | 3031±417 | 3282±263 | 3023±284 | 0.53 | 0.55 | 0.68 |
| IL-6 | 73±9 ^a^ | | 138±26 ^b^ | 183±42 ^b^ | 86±17 ^a^ | 0.81 | 0.92 | <0.01 |
| TGF-β | 250±18 | | 199±15 | 224±31 | 232±14 | 0.98 | 0.32 | 0.09 |
| TNF-α | 49±20 | | 102±22 | 78±21 | 92±22 | 0.41 | 0.02 | 0.16 |
| IL-10 | 57±12 | | 96±32 | 69±12 | 91±10 | 0.27 | 0.19 | 0.74 |
| IL-4 | 5.2±1.1 | | 8.4±2.4 | 8.7±2.1 | 11.3±3.0 | 0.07 | 0.12 | 0.60 |
| IL-13 | 9.4±1.9 | | 13.9±2.5 | 12.9±3.2 | 9.8±1.3 | 0.67 | 0.35 | 0.18 |
| IFN-γ | 270±215 | | 612±300 | 321±119 | 346±122 | 0.07 | 0.04 | 0.23 |
| **Unstimulated (pg/ml)** | | |  |  |  |  |  |  |
| TNF-α | 17±1 | | 18±2 | 25±5 | 21±2 | 0.58 | 0.70 | 0.87 |
| IL-10 | 42±6 ^b^ | | 59±11 ^b^ | 52±10 ^b^ | 24±3 ^a^ | 0.07 | 0.05 | <0.01 |

^1^ Data is presented in mean ± standard error of mean. Means within a row without a common superscript letter are significantly different, *P* < 0.05. Abbreviations: SD, suckling diet; WD, post-weaning diet; ARA, arachidonic acid; DHA, docosahexaenoic acid; PMAi, phorbol-myristate-acetate and ionomycin; IL, interleukin; TGFβ, transforming growth factor-β; TNFα, tumour necrosis factor-α; IFNγ, interferon-γ; SEM, standard error of mean; SD, suckling diet; WD, post-weaning diet.

^2^ Indicates P-value for the main effect of the SD in mixed model.

^3^ Indicates P-value for the main effect of the WD in mixed model.

^4^ Indicates interaction between maternal SD and offspring WD.

Supplementary Table 5. The effect of supplementing suckling diet and post-weaning diet on *ex-vivo* cytokine production by mesenteric lymph node cells after stimulation with mitogens ^1^

| Suckling diet | Control | Control | ARA+DHA | ARA+DHA | *P*-SD^2^ | *P*-WD^3^ | *P*-SD × WD^4^ |
| --- | --- | --- | --- | --- | --- | --- | --- |
| Post-weaning diet | Control | ARA+DHA | Control | ARA+DHA |  |  |  |
|  | Mean ± SEM (n=6) | Mean ± SEM (n=5) | Mean ± SEM (n=8) | Mean ± SEM (n=7) |  |  |  |
| **Lipopolysaccharide (pg/ml)** | |  |  |  |  |  |  |
| IL-10 | 69±22 ^a^ | 29±9 ^b^ | 23±4 ^b^ | 25±7 ^b^ | 0.21 | 0.29 | 0.03 |
| **PMAi (pg/ml)** | |  |  |  |  |  |  |
| TGF-β | 229±10 | 244±12 | 193±41 | 292±50 | 0.99 | 0.16 | 0.25 |
| IL-2 | 3412±1708 | 4807±1823 | 3897±1666 | 3078±1400 | 1.00 | 1.00 | 0.23 |
| IL-10 | 56±21 | 80±28 | 52±19 | 57±26 | 0.70 | 0.42 | 0.32 |
| TNF-α | 149±73 | 158±71 | 126±43 | 134±41 | 0.37 | 0.31 | 0.46 |

^1^ Data is presented in mean ± standard error of mean. Means within a row without a common superscript letter are significantly different, *P* < 0.05. Abbreviations: ARA, arachidonic acid; DHA, docosahexaenoic acid; SEM, standard error of mean; SD, suckling diet; WD, post-weaning diet; PMAi, phorbol-myristate-acetate and ionomycin; IL, interleukin; TGFβ, transforming growth factor-β; TNFα, tumour necrosis factor-α.

^2^ Indicates P-value for the main effect of the SD in mixed model.

^3^ Indicates P-value for the main effect of the WD in mixed model.

^4^ Indicates interaction between maternal SD and offspring WD.

Supplementary Table 6: Description of immune cells phenotype used to identify immune cell subpopulations from lymphoid tissues collected from 8-week-old Brown Norway rat offspring ^1^

| **Phenotype** | **Cell types** | **Tissues** |
| --- | --- | --- |
| **T cells** |  |  |
| CD3+ | Total T cells | Spleen, MLN and PP |
| TCRαβ | T cells with αβ-TCR | Spleen and MLN |
| CD3+CD8+ | CTL | Spleen, MLN and PP |
| CD3+CD8+CD25+ | Activated CTL | Spleen, MLN and PP |
| CD3+CD4+ | Th cells | Spleen, MLN and PP |
| CD3+CD4+CD25+ | Activated Th cells | Spleen, MLN and PP |
| CD3+CD4+CD25+FoxP3+ | Treg cells | Spleen and MLN |
| CD8+CD4-CD152+ | CTLA-4 on CTL | Spleen and MLN |
| CD8+CD4-CD28+ | Co-stimulatory marker on CTL | Spleen and MLN |
| CD4+CD8-CD152+ | CTLA-4 on Th | Spleen and MLN |
| CD4+CD8-CD28+ | Co-stimulatory marker on Th | Spleen and MLN |
| CD27+ | Total memory lymphocytes | Spleen and MLN |
| **B cells** |  |  |
| CD45RA+ | Total B cells and naïve leukocytes | Spleen, MLN and PP |
| OX12+ | B cell subtype with | Spleen and MLN |
| CD45RA+IgG+ | B cell subtype with IgG | Spleen, MLN and PP |
| CD45RA+IgE+ | B cell subtype with IgE | Spleen, MLN and PP |
| CD45RA+IgA+ | B cell subtype with IgA | Spleen, MLN and PP |
| **Innate immune cells** |  |  |
| CD3-CD161+ | Natural Killer cells | Spleen |
| CD3+CD161+ | Natural Killer T cells | Spleen |
| OX6+ | APC | Spleen, MLN and PP |
| OX62+ | DC | Spleen, MLN and PP |
| CD68+ | Tissue Macrophages | Spleen and PP |
| CD11+ | Macrophages and granulocytes | Spleen and PP |
| CD284+ | TLR4 on macrophage, DC and B cells | Spleen and PP |

Abbreviation: MLN, mesenteric lymph nodes; PP, peyer’s patches; CTL Cytotoxic T cells; αβ-TCR, αβ-T cell receptors; Th, Helper T cells; Treg, regulatory T cells; APC, antigen presenting cells; CTLA-4, Cytotoxic T-Lymphocyte Associated Protein 4; TLR4-4, toll like receptor-4; DC, dendritic cells

^1^ The gating strategy for main cell types has been reported in supplementary figure 1.
